# Characterization of indigenous populations of cannabis in Iran: a morphological and phenological study

**DOI:** 10.1186/s12870-024-04841-y

**Published:** 2024-02-29

**Authors:** Mehdi Babaei, Hossein Nemati, Hossein Arouiee, Davoud Torkamaneh

**Affiliations:** 1 Department of Horticultural Sciences, Ferdowsi University of Mashhad, Azadi Square, Mashhad, 9177948974 Razavi Khorasan Iran; 2 Département de Phytologie, Université Laval, Rue de l’Université, Québec City, Québec G1V 0A6 Canada; 3 Institut de Biologie Intégrative et des Systèmes (IBIS), Université Laval, Rue de l’Université, Québec City, Québec G1V 0A6 Canada; 4 Centre de recherche et d’innovation sur les végétaux (CRIV), Rue de l’Agriculture , Université Laval, Québec City, Québec G1V 0A6 Canada; 5 Institute Intelligence and Data (IID), Rue de l’Agriculture Québec City, Université Laval, Québec City, Québec G1V 0A6 Canada

**Keywords:** Phenotyping, Germplasm, Heritability, Hemp, Landraces, Relative growth rate, Descriptors, Spatial analysis, Indirect breeding

## Abstract

**Background:**

Cannabis is a historically, culturally, and economically significant crop in human societies, owing to its versatile applications in both industry and medicine. Over many years, native cannabis populations have acclimated to the various environments found throughout Iran, resulting in rich genetic and phenotypic diversity. Examining phenotypic diversity within and between indigenous populations is crucial for effective plant breeding programs. This study aimed to classify indigenous cannabis populations in Iran to meet the needs of breeders and breeding programs in developing new cultivars.

**Results:**

Here, we assessed phenotypic diversity in 25 indigenous populations based on 12 phenological and 14 morphological traits in male and female plants. The extent of heritability for each parameter was estimated in both genders, and relationships between quantitative and time-based traits were explored. Principal component analysis (PCA) identified traits influencing population distinctions. Overall, populations were broadly classified into early, medium, and late flowering groups. The highest extent of heritability of phenological traits was found in Start Flower Formation Time in Individuals (SFFI) for females (0.91) Flowering Time 50% in Individuals (50% of bracts formed) (FT50I) for males (0.98). Populations IR7385 and IR2845 exhibited the highest commercial index (60%). Among male plants, the highest extent of Relative Growth Rate (RGR) was observed in the IR2845 population (0.122 g.g^− 1^.day^− 1^). Finally, populations were clustered into seven groups according to the morphological traits in female and male plants.

**Conclusions:**

Overall, significant phenotypic diversity was observed among indigenous populations, emphasizing the potential for various applications. Early-flowering populations, with their high RGR and Harvest Index (HI), were found as promising options for inclusion in breeding programs. The findings provide valuable insights into harnessing the genetic diversity of indigenous cannabis for diverse purposes.

**Supplementary Information:**

The online version contains supplementary material available at 10.1186/s12870-024-04841-y.

## Introduction

Cannabis (*Cannabis sativa* L.), a predominantly dioecious diploid (2n = 20), is one of the earliest domesticated plants, cultivated worldwide for various purposes including fiber production, seed cultivation, oil extraction, and harnessing its distinct psychoactive and medicinal properties [1]. Recently, legalization in different countries has spurred a surge in recognition and research on cannabis [2]. Cannabis is known to produce over 545 potentially bioactive secondary metabolites, including more than 120 cannabinoids, various flavonoids, and a plethora of terpenes. In many regions across the world (e.g., Canada, the USA, and Europe), cannabis is commonly divided and regulated based on the level of the psychoactive cannabinoid (i.e., delta-9-tetrahydrocannabinol (D^9^THC)) produced in the plant. Plants with less than 0.3% are regulated as hemp, while those with 0.3% or more are considered drug-type cannabis). The global legal cannabis market is undergoing unprecedented growth, with projections indicating that it will soar to a remarkable $102 billion by 2028, nearly doubling its current value of $51 billion [3]. This growth positions cannabis as one of the most economically significant crops on a global scale. Despite this rapid commercial expansion, the fundamental biology of cannabis remains unknown, mainly due to years of prohibition.

As a member of the Cannabaceae family, cannabis can be found in a variety of habitats and at different altitudes. It is suggested that cannabis may have originated from the foothills of the Himalayan Alps before spreading to diverse regions across the globe over the past millennium [4]. This extensive dispersal led to the evolution of diverse cannabis populations through a combination of natural and artificial selection in response to various environments in which they were introduced. However, these gene pools have largely remained untapped in comparison to other crops, primarily due to the complex legal history surrounding cannabis. Until very recently, cannabis breeding was predominantly conducted within clandestine operations, relying on a severely limited genetic pool. These clandestine efforts often focused exclusively on high-THC drug-type plants, utilizing undocumented methods and lacking access to modern technologies [5]. In plant breeding, the initial step involves inbreeding to stabilize desired traits. This is a challenge in cannabis as cannabis plants are predominantly dioecious, meaning male and female flowers are on separate plants [6]. However, in the cannabis industry, unfertilized female plants are preferred as they produce the most cannabinoids [7]. The absence of conventional breeding programs has led to a shortage of uniform inbred seeds, forcing producers to heavily rely on clonal propagation. Despite the assumption that cuttings taken from mother plants will provide uniform plants, recent studies show that they change over time, becoming less vigorous and producing lower levels of cannabinoids [8]. Advanced DNA sequencing techniques have unveiled a significant intra-plant genetic diversity within a single mother plant [9]. The stabilized cultivars derived from the inbreeding process represent a unique opportunity for cannabis growers to not only produce uniform products but also explore new combinations of cannabis genetics to develop disease resistance, new flavors and aromas, alter cannabinoid levels, and boost total yields [10]. The challenges of prohibition and a limited gene pool, particularly skewed towards high-THC varieties without considering resilience against pests and diseases, contribute to a bottleneck in genetic diversity [11]. With the current paradigm shift in the legal status of cannabis, there is both the opportunity and a pressing need to delve into larger genetic pools and harness the existing genetic and phenotypic diversity. This marks a fundamental stride towards realizing success in modern cannabis breeding, with applications spanning agriculture and medicine [12, 13].

Plant genetic materials found in natural reserves, including landraces, serve as the primary sources of genetic diversity. These reserves are also instrumental in providing novel traits used in breeding [14] and play a crucial role in breeding programs to adapt to changes in environments and evolving market demand [15, 16]. Investigating these resources facilitates the organized management of genetic reserves, efficient genotype selection, and the judicious exploitation of diversity [17]. Iran is recognized as one of the significant genetic reservoirs of cannabis due to being one of its prominent habitats. Flora Iranica documents the widespread distribution of wild cannabis across diverse regions of Iran [18–20]. Over many years, these native cannabis populations have acclimated to the various climates found throughout Iran, resulting in a rich genetic and phenotypic diversity. This diversity holds significant potential for industrial and medicinal applications, as well as long-term integration into breeding programs for different traits such as disease resistance. This is important, as many commercial cultivars that are bred and used globally can trace their origins back to this region [21].

Recently, several studies have focused on characterizing agronomic, morphological, and phenological traits in cannabis. However, these investigations were primarily restricted to commercial genotypes and predominantly centered on drug-type cannabis varieties. For instance, a comprehensive characterization of 13 morphophysiological and phenological traits in 121 commercial genotypes of cannabis plants under controlled conditions, revealing that significant phenotypic variation persists despite a decrease in genetic diversity within commercial resources [22]. Similarly, another research conducted phenotypic characterization of 176 commercial cannabis genotypes under a greenhouse setting, focusing on 13 agronomic and morphological traits. Their study unveiled significant variations among the population in several traits indicating the promising perspective of cannabis breeding [6]. Additionally, previous research delved into the genetics of hemp plants, especially in hybrids production, using 23 diverse genetic “families” to investigate the inheritance of morphological characteristics and proposing a new classification method for the architecture of cannabis plants [23].

Understanding the morphological and phenological traits of cannabis is fundamental to unlocking its potential for breeding and cultivation. In 1998, reported a comprehensive description of the phenological events in cannabis [24]. These stages were systematically defined, encompassing the entire life cycle of the cannabis plant, from the initial emergence of the radicle to the eventual maturation of seeds. What sets this descriptor apart is its consideration of the phenological stages in the context of male, female, and monoecious plants, providing a valuable framework for a deeper understanding of cannabis development and reproduction. The recognition and analysis of morphological and phenological traits unlocks a deeper understanding of indigenous plant populations and plays a prominent role in plant breeding. Morphological traits, in particular, offer valuable insights into the physical characteristics and structures of cannabis plants, while phenological traits encapsulate the timing and progression of key events in the plant’s life cycle. This dual approach offers an indispensable foundation for improving plant performance, yield, and adaptability, ultimately contributing to the sustainability and success of this industry [23].

In this study, we collected and assessed 25 indigenous populations from Iran under controlled environmental conditions. Our evaluation encompasses 26 key phenological and morphological traits, which enabled the classification of these plants based on their suitability for breeding programs. This study not only advances our understanding of indigenous cannabis populations but also highlights their potential for integration into contemporary breeding initiatives.

## Results

### Trends in phenological parameters in male and female plants in different populations

The germination rate ranged from 30.7 to 100%, with a corresponding rate ranged from the highest 21 seeds/day to the slowest at 4 seeds/day. Additional details of the germination test are provided in Fig S1.

**Fig. 1:**
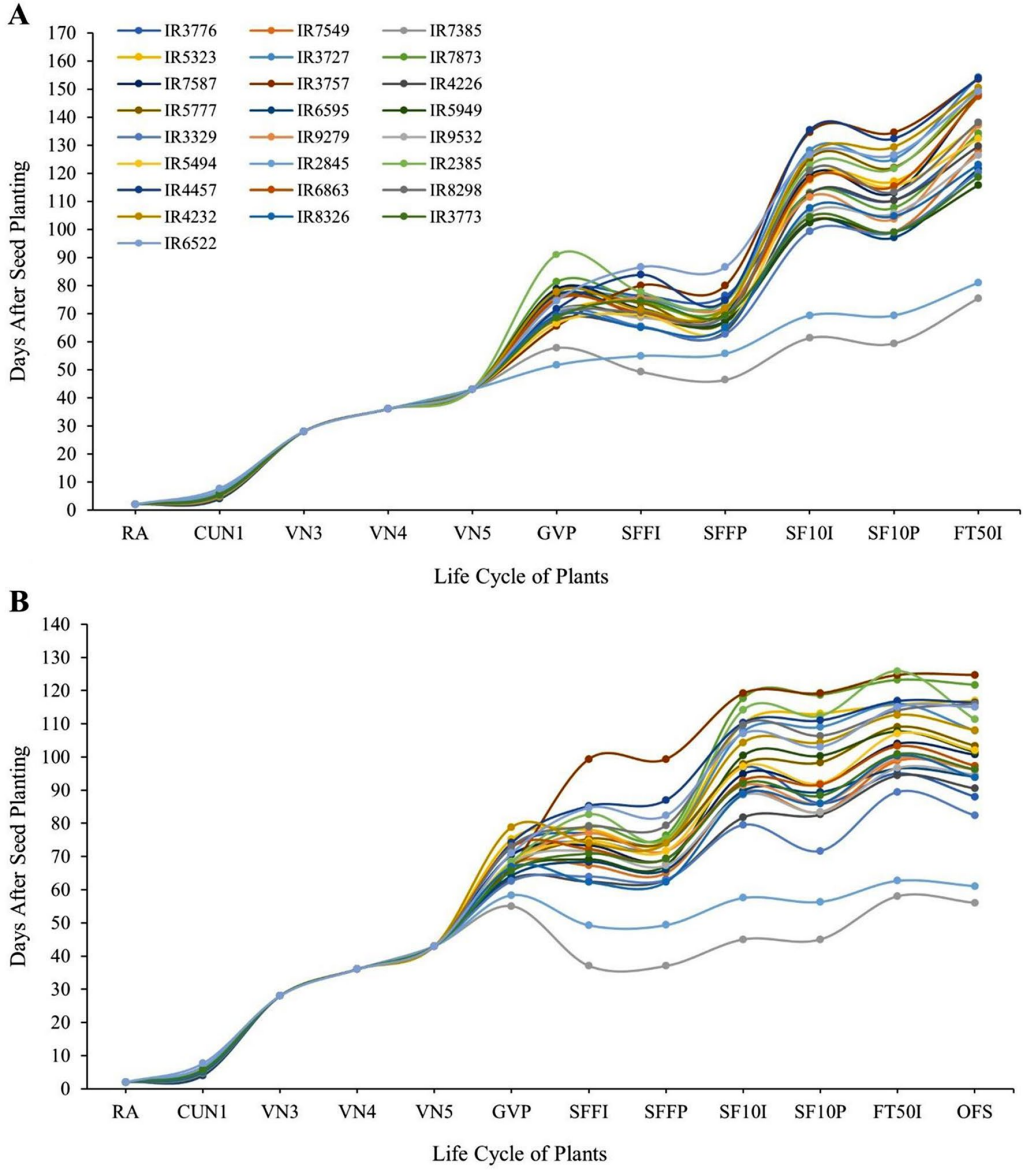
Plant (female (**A**) and male (**B**)) life patterns in 25 native cannabis populations of Iran. RA: Radicle Apparent, CUN1: Cotyledons Unfolded (1st node), VN3: Vegetative Stage (2nd leaf pair), VN4: Vegetative Stage (3rd leaf pair), VN5: Vegetative Stage (4th leaf pair), GVP: GV Point, SFFI: Start Flower Formation Time in Individuals, SFFP: Start Flower Formation Time in 50% Population, SF10I: Start 10% Flowering Time in Individuals (10% of bracts formed), SF10P: Start 10% Flowering Time in 50% Population (10% of bracts formed), FT50I: Flowering Time 50% in Individuals (50% of bracts formed), OFS: First Opened Staminate Flowers

We observed a consistent pattern in the phenological parameters of male and female plants across all populations (Fig. 1), from the Radicle Apparent stage (RA) to the appearance of the fifth node (VN5), which typically occurred within 43 days after germination. As shown in Fig. 1, the transition in phyllotaxis from opposite to alternate (GV Point), in both male and female plants, occurred after the plants entered the reproductive phase. In certain populations (IR7549, IR7385, IR7873, IR7587, IR6595, IR3329, IR2385, IR6863, IR4232 and IR8326 in female plants; IR7385, IR2845, IR4232 and IR8326 in male plants), this transition occurred after the appearance of solitary flowers (SFFI and SFFP). The remaining populations exhibited phyllotaxis changes before reaching the SF10I and SF10P stages.

The examination of diversity and uniformity in the SFFI and SF10I stages revealed a maximum six-day difference in female plants between SFFI and SFFP, while an eight-day difference was detected between SF10I and SF10P, representing the longest time interval. In male plants, the most extended time difference (8 days) was observed between SFFI and SFFP, while a 10-day difference was noted between SF10P and SF10I. It is worth mentioning that SFFI and SF10I were evaluated for the entire population, while SFFP and SF10P were recorded for 50% of individuals. FT50I, marking the last phenological stage, took 26 days in the early-flowering female population, with the longest difference observed at 78 days (Fig. 1A). In early-flowering male plants, this difference was 13 days on average, with the highest variation reaching 44 days (Fig. 1B). The OFS was recorded around the time of SFFI and generally coincided with SF10I, reflecting the time needed for pollen maturation until anther sac rupture.

**Fig. 2:**
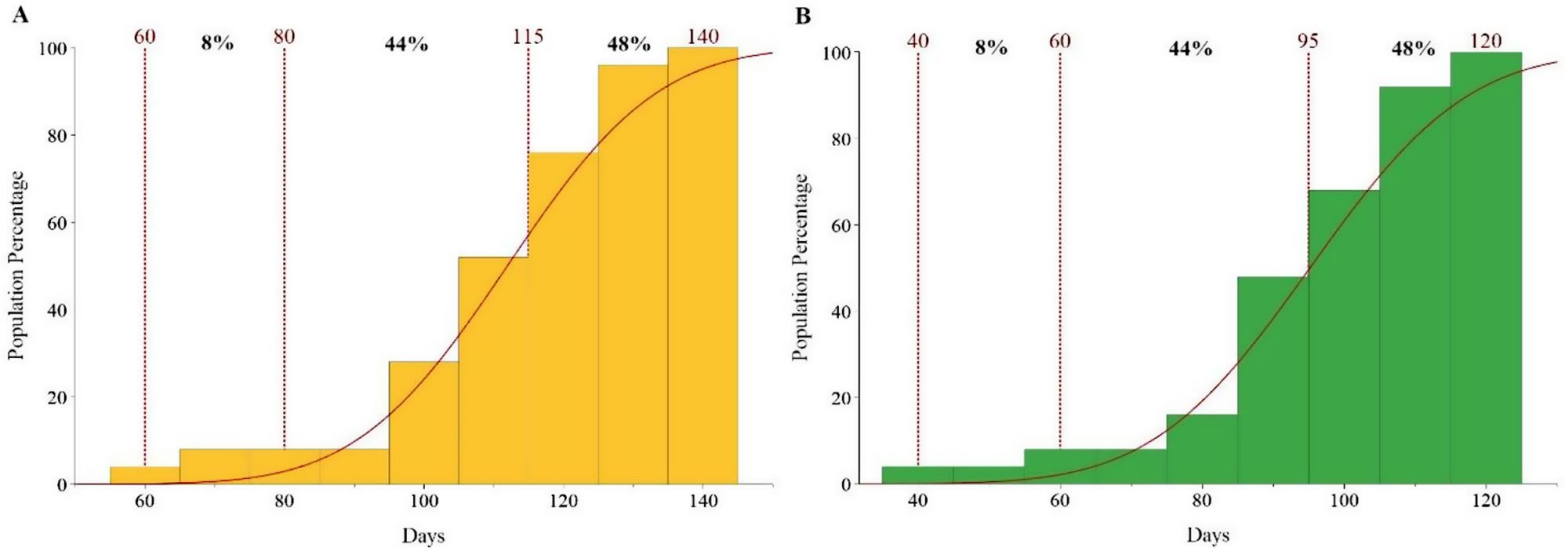
Division of 25 native Cannabis populations of Iran based on Start 10% Flowering Time in Individuals (10% of bracts formed) (SF10I). in female (**A**) and male (**B**) plants

According to the general phenological classification (Fig. 2) using SF10I, both male and female plants were classified into three groups. In female plants, the early-flowering group included those with SF10I appearance between 60 and 80 days, making up 8% of the total population. The second group included populations of female plants classified as medium-flowering plants with SF10I appearance between 80 and 115 days, accounting for 44% of the entire population. The third group classified as late-flowering involved SF10I appearance between 115 and 140 days, representing 44% of the total population (Fig. 2A). For male plants, the early-flowering group constituted 8% of the entire population, with SF10I appearance occurring in the time frame of 40 to 60 days. The medium-flowering group involved SF10I appearance within the range of 60–95 days, also constituting 44% of the entire population, similar to the female plants. Finally, the late-flowering male plants constituted 48% of the total population, with SF10I appearing between 95 and 120 days (Fig. 2B).

### Broad-sense heritability among phenological parameters

The extent of broad-sense heritability (H^2^) was assessed across all populations for both male and female plants, revealing a diverse range of values among phenological parameters (Table 1). The highest H^2^ value, 0.9, was found in the SFFI and SFFP parameters for the female plants, signifying a 32.96% reduction in the FT50I parameter. The extent of H^2^ in male plants was found to be the same (0.96) in the SFFI and SFFP parameters, while another set of parameters, including SF10I, SF10P, and OFS exhibited the same H^2^ value of 0.97. In the case of male plants, the FT50I parameter had the highest H^2^ at 0.98, resulting in a 23.4% reduction in the GVP parameter.

**Table 1:** Broad‑sense heritability (H^2^) for phenological traits in 25 native cannabis populations of Iran

Parameter	Female	Male
GVP	0.80	0.75
SFFI	0.91	0.96
SFFP	0.90	0.96
SF10I	0.73	0.97
SF10P	0.76	0.97
FT50I	0.61	0.98
OFS	-	0.97

*Abbreviations *GVP: GV Point, SFFI: Start Flower Formation Time in Individuals, SFFP: Start Flower Formation Time in 50% Population, SF10I: Start 10% Flowering Time in Individuals (10% of bracts formed), SF10P: Start 10% Flowering Time in 50% Population (10% of bracts formed), FT50I: Flowering Time 50% in Individuals (50% of bracts formed), OFS: First Opened Staminate Flowers

### Population classification using phenological traits

**Fig. 3:**
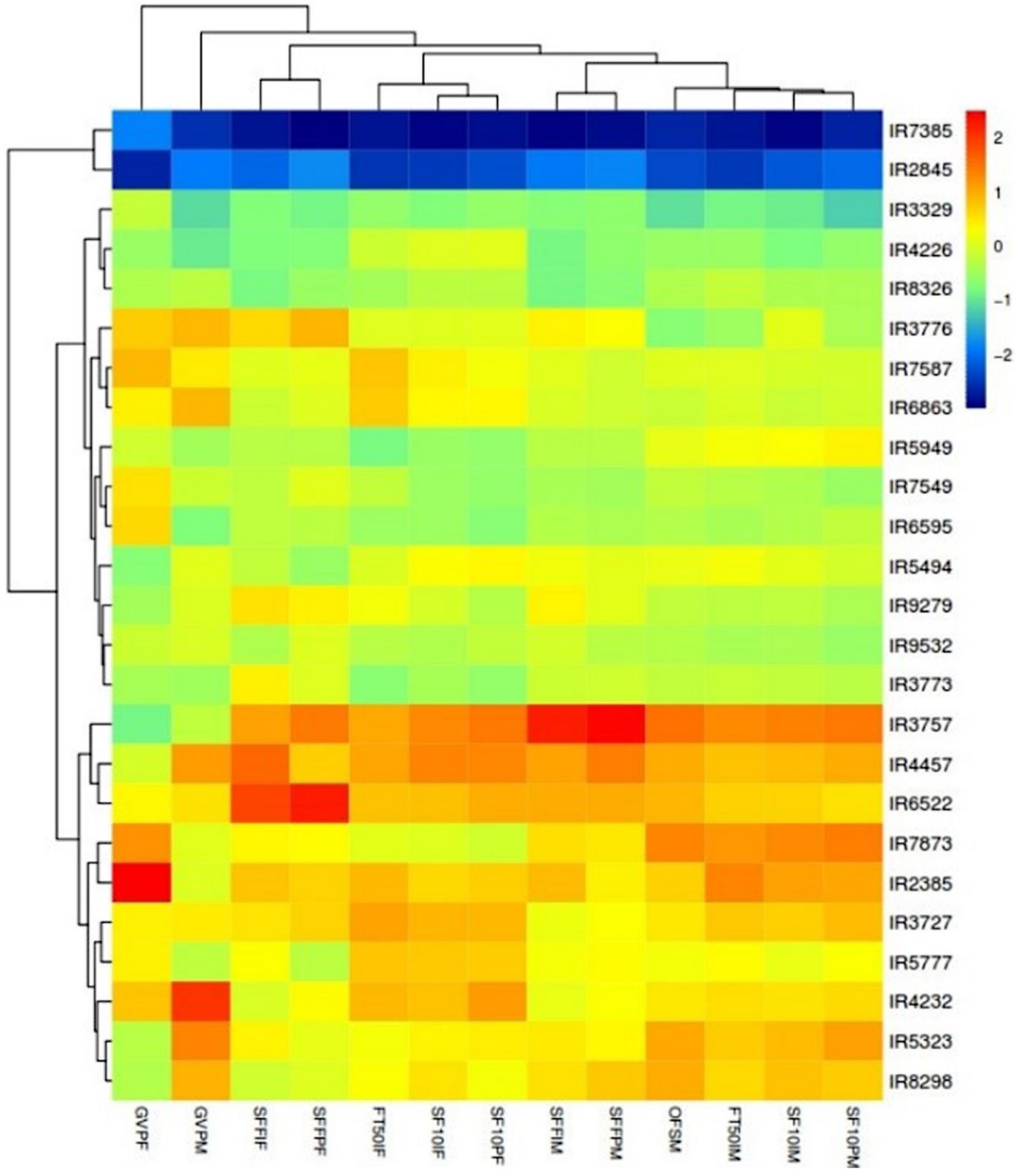
Heatmap based on the data of phenological traits in 25 native cannabis populations of Iran (male and female). GVP: GV Point, SFFI: Start Flower Formation Time in Individuals, SFFP: Start Flower Formation Time in 50% Population, SF10I: Start 10% Flowering Time in Individuals (10% of bracts formed), SF10P: Start 10% Flowering Time in 50% Population (10% of bracts formed), FT50I: Flowering Time 50% in Individuals (50% of bracts formed), OFS: First Opened Staminate Flowers. M: Male and F: Female

The phenological traits with the highest variances were selected, and their mean values, standardized for both male and female plants, were used for classification. The populations were classified into five groups based on their flowering behavior (Fig. 3). The first group, consisting of early-flowering populations, had the lowest values across all parameters and included the IR7385 and IR2845 populations. The second and third groups, including medium-flowering populations, differed mainly in the timing of GV Point and SFFP appearance. The second group had three populations, while the third group had ten populations. The fourth group, with three populations, exhibited the longest time for SFFI and SFFP, which was consistent between male and female plants. The fifth group included seven populations, featuring the most extended time for GV Point in both male and female plants, distinguishing it from the fourth group.

### Morphological parameters of male and female plants in different populations

**Fig. 4:**
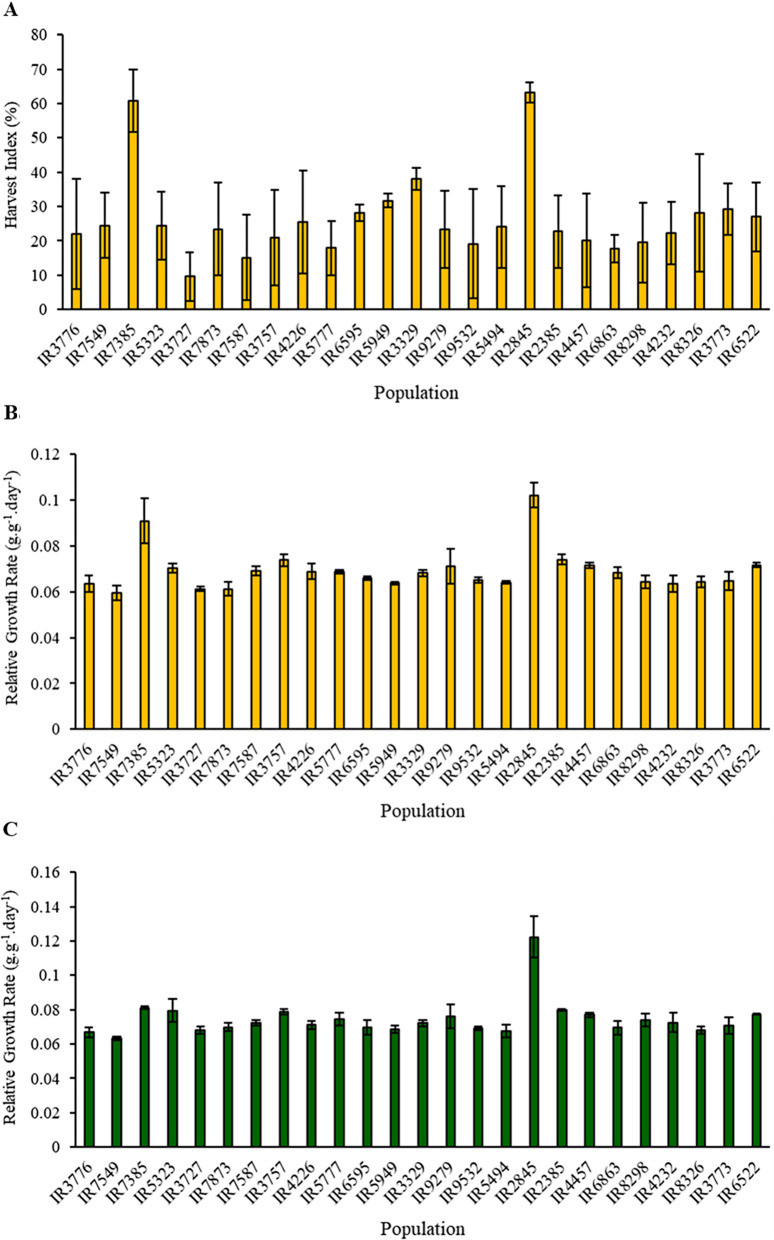
Comparison of 25 native cannabis populations of Iran based on Harvest Index (Commercial Index) (HI) in female plants (**A**), LSD _0.01_= 23.7), and Relative Growth Rate (RGR) in female plants (**B**), LSD _0.01_= 0.0072) and male plants (**C**), LSD _0.01_= 0.0087)

Fourteen morphological traits were assessed and analyzed (Table 2). Apart from the two functional traits, DWF and FWF in male plants, significant differences were observed across all the examined traits in various populations of both male and female plants. A wide range and high coefficient of variation (CV) values were observed. The trait with the highest CV was HI (56.95%), which was primarily associated with biomass. Conversely, the traits NNH and RGR displayed the lowest CV values (10.81 (female); 14.55 (male) and 13.85 (female); 15.28 (male), respectively). Based on the mean comparison related to HI in female plants (Fig. 4A), populations IR7385 and IR2845 had the highest commercial index values among the populations (60%). In contrast, population IR3727 had the lowest commercial index value (9.5%). Further exploration of RGR values (Fig. 4B and C) showed that the IR7385 and IR2845 populations had the highest RGR values, attributed to their shorter growth periods and early flowering nature. For female plants, RGR values reached 0.091 and 0.102 g.g^− 1^.day^− 1^, respectively. In the case of male plants, the IR2845 population achieved the highest RGR value at 0.122 g.g^− 1^.day^− 1^. Additional details on the variance analysis are available in Tables S1 and S2.

**Table 2:** Summary statistics based on 14 morphological traits in 25 native cannabis populations of iran, differentiated by female and male plants

Trait	Unit	Gender	Mean ± SD	Range	CV%	Significance
LMI	cm	Female	26.6 ± 7.05	33.7	26.5	***
		Male	33.03 ± 10.74	39.3	32.51	***
PT	1 to 4	Female	3.22 ± 0.62	2	19.31	**
		Male	2.95 ± 0.74	3	25.16	***
SDH	mm	Female	14.4 ± 2.8	17.2	19.23	***
		Male	11.7 ± 2.63	13.3	22.5	***
HH	cm	Female	155.1 ± 35.66	189.2	22.99	***
		Male	153.5 ± 38.22	207.9	24.89	***
NNH	number	Female	28.75 ± 3.11	18	10.81	***
		Male	27.8 ± 4.05	18.6	14.55	***
LIMTH	cm	Female	12.66 ± 3.5	18.2	27.58	***
		Male	13.05 ± 3.27	16.9	25.06	***
NLS	number	Female	23.7 ± 4.76	24.5	20.08	***
		Male	22.26 ± 5.67	32	25.46	***
HGV	cm	Female	58.3 ± 19.05	100	32.66	***
		Male	53.25 ± 19.62	115	36.84	**
FWF	g	Female	88.35 ± 37	160.5	41.88	ns
		Male	28.99 ± 13.25	64.3	45.71	***
DWF	g	Female	26.77 ± 11.05	44.9	41.27	ns
		Male	10.33 ± 4.58	18.65	44.32	***
TFW	g	Female	278.38 ± 63.74	316.6	22.9	***
		Male	151.34 ± 74.59	332.65	49.28	***
TDW	g	Female	115.43 ± 32.08	159.12	27.79	**
		Male	62.22 ± 29.12	129.78	46.8	***
HI	%	Female	26.33 ± 15	68.22	56.95	**
		Male	-	-	-	-
RGR	g.g^− 1^.day^− 1^	Female	0.069 ± 0.0095	0.05	13.85	***
		Male	0.074 ± 0.0113	0.074	15.28	***

Mean ± standard deviation; CV: coefficient of variation; range = maximum value − minimum value; ns, *, ** and *** indicate significant differences at not significant, *P* ≤ 0.05, 0.01 and 0.001, respectively. Abbreviations; DWF: Dry Weight of Flowers, FWF: Fresh Weight of Flowers, HGV: Height to GV Point, HH: Height on Harvest day, HI: Harvest Index, LIMTH: Length of Internode in the Middle Third of the main stem on Harvest day, LMI: Length of Main Inflorescence, NLS: Number of Lateral Shoot, NNH: Number of Nodes on the main stem on Harvest day, PT: Plant Type (1 to 4), RGR: Relative Growth Rate, SDH: Stem Diameter on Harvest day, TDW: Total Dry Weight, TFW: Total Fresh Weight

**Fig. 5:**
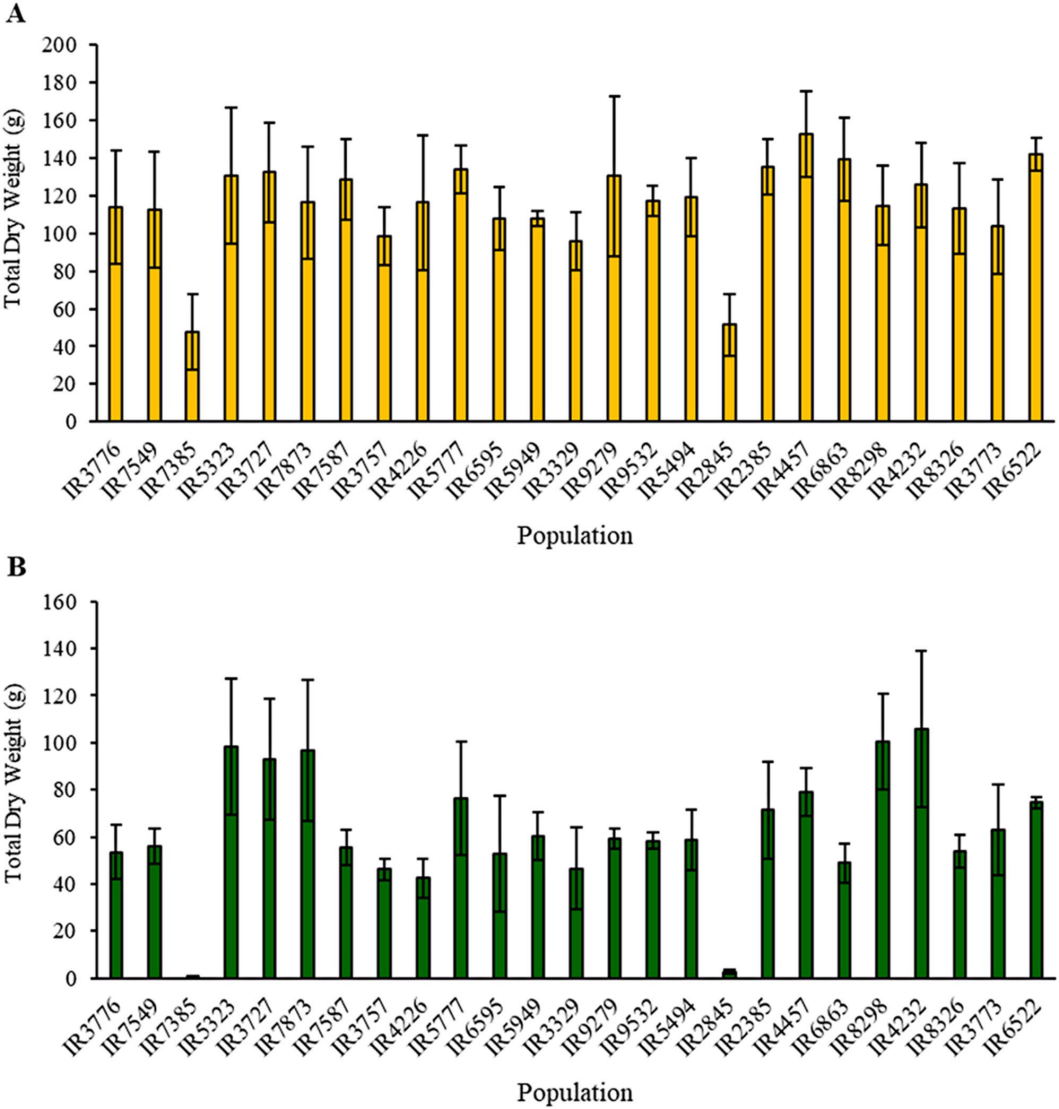
Comparison of 25 native cannabis populations of Iran based on Total Dry Weight (TDW) in female plants (**A**), LSD _0.01_= 56.4) and male plants (**B**), LSD _0.01_= 34.9)

Finally, comparison of the mean TDW in female plants (Fig. 5A) showed that, within a range overlapping with standard deviations, the IR4457 population was highlighted as an example with the highest value (152.77 g). This value significantly exceeded the TDW of the IR7385 and IR2845 populations by 68%. Notably, in male plants including IR4232, IR8298, IR7873, IR3727, and IR5323 displayed TDW values in the range of 92 to 106 g. In contrast, populations IR7385 and IR2845 had the lowest TDW values, recording merely 1.05 and 2.79 g, respectively (Fig. 5B).

### Broad-sense heritability of morphological parameters

The estimated broad-sense heritability values for fourteen morphological traits are displayed in Table 3. The values ranged from 0.23 (for FWF) to 0.95 (for RGR) in female plants and from 0.56 (for LIMTH) to 0.99 (for RGR) in male plants. In general, male plants showed higher estimated H^2^ values than female plants across all morphological traits, except for HGV and LIMTH, where the values were higher in female plants than in males. The H^2^ values in females varied in functional traits; DWF and FWF, which are related to bud traits, showed lower values than the other two functional parameters, TDW and TFW, which are linked to the biomass of the entire plant. Generally, H^2^ value for TDW trait in male plants tends to be lower than in female plants. This is because male plants allocate a larger portion of their resources towards pollen production and less towards vegetative growth compared to females.

**Table 3:** Broad‑sense heritability (H^2^) for morphological traits in 25 native cannabis populations of Iran

Parameter	Female	Male
DWF	0.33	0.87
FWF	0.23	0.92
HGV	0.89	0.69
HH	0.92	0.96
HI	0.63	-
LIMTH	0.87	0.56
LMI	0.76	0.97
NLS	0.88	0.97
NNH	0.81	0.94
PT	0.76	0.95
RGR	0.95	0.99
SDH	0.81	0.95
TDW	0.65	0.94
TFW	0.90	0.96

*Abbreviations *DWF: Dry Weight of Flowers, FWF: Fresh Weight of Flowers, HGV: Height to GV Point, HH: Height on Harvest day, HI: Harvest Index, LIMTH: Length of Internode in the Middle Third of the main stem on Harvest day, LMI: Length of Main Inflorescence, NLS: Number of Lateral Shoot, NNH: Number of Nodes on the main stem on Harvest day, PT: Plant Type (1 to 4), RGR: Relative Growth Rate, SDH: Stem Diameter on Harvest day, TDW: Total Dry Weight, TFW: Total Fresh Weight

### Population classification using morphological traits

**Fig. 6:**
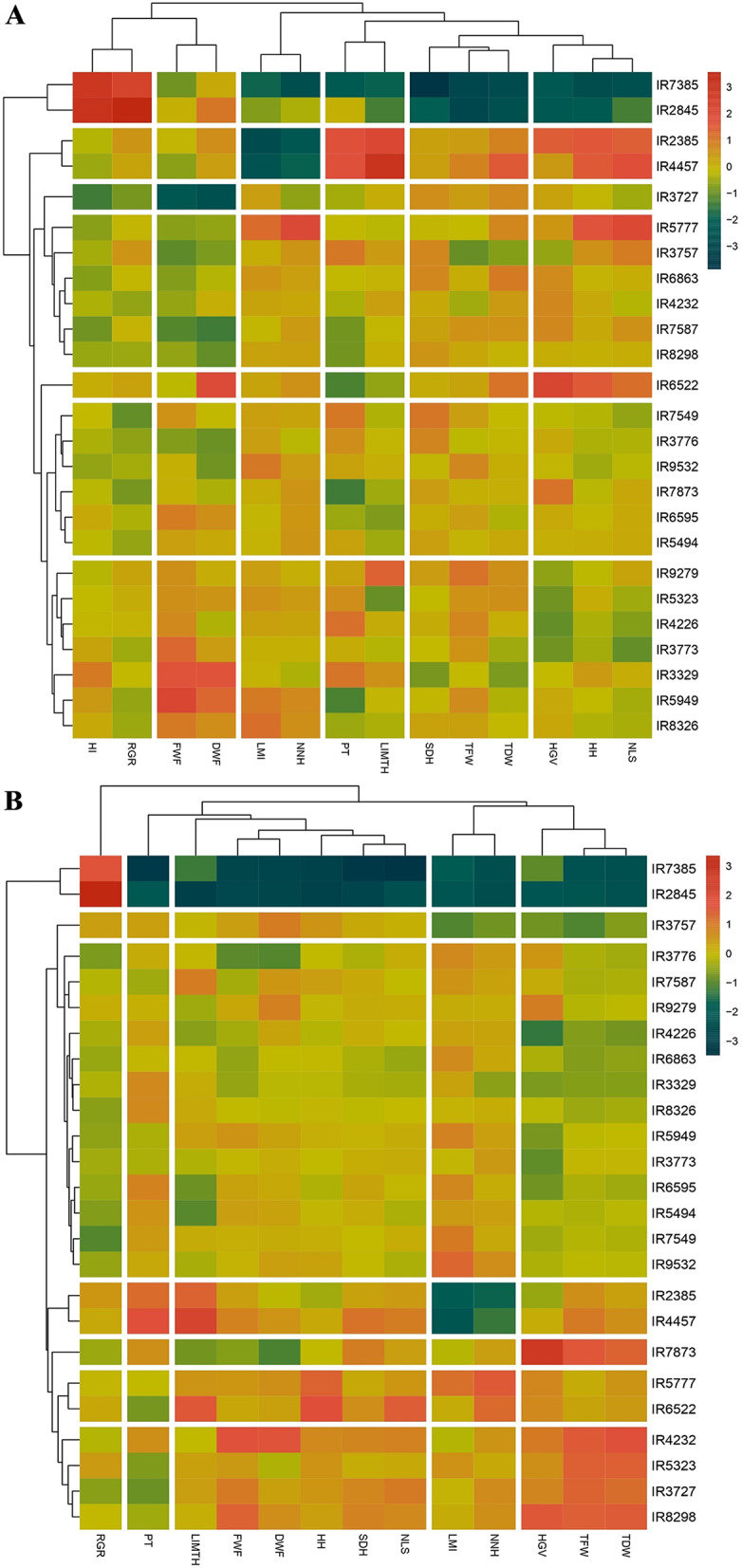
Heatmap based on morphological data (14 and 13 traits for female and male plants, respectively) in 25 native cannabis populations of Iran in female plants (**A**) and male plants (**B**). Abbreviations; LMI: Length of Main Inflorescence, PT: Plant Type (1 to 4), SDH: Stem Diameter on Harvest day, HH: Height on Harvest day, NNH: Number of Nodes on the main stem on Harvest day, LIMTH: Length of Internode in the Middle Third of the main stem on Harvest day, NLS: Number of Lateral Shoot, HGV: Height to GV Point, FWF: Fresh Weight of Flowers, DWF: Dry Weight of Flowers, TFW: Total Fresh Weight, TDW: Total Dry Weight, HI: Harvest Index, RGR: Relative Growth Rate

The female and male plants within the examined populations were subjected to clustering using Heatmap, based on 14 and 13 morphological traits, respectively (Fig. 6). In the female plants (Fig. 6A), the populations were classified into seven distinct groups, while the morphological traits were partitioned into six distinct classes. It is noteworthy that two of these clusters included only one population each (IR3727 and IR6522 in one, and IR7385 and IR2845 in another). The remaining two clusters included six populations each, and the last cluster encompassed seven populations. Following the classification of these traits, it was found that HI and RGR were part of the same group, while parameters such as TDW, TFW, and SDH were placed in different groups.

Similarly male plants were grouped into seven distinct clusters. Populations including IR7385 and IR2845 were clustered together. However, for the other five clusters, a different arrangement was observed. For instance, populations IR3757 and IR7873 were placed in different clusters. On the other hand, populations IR5777 and IR6522 were placed in the same cluster. Of the last two clusters, one contained four populations, while the other entailed thirteen populations, making it the largest cluster of male plants (Fig. 6B). The parameters were grouped somewhat differently for male plants compared to female plants. Generally, they were classified into five different groups, with PT and RGR each having their own group. Parameters including TDW, TFW, and HGV were grouped together, specified using a vertical white line in Fig. 6B.

### Understanding the correlation and relation between morphological and phenological traits

**Fig. 7:**
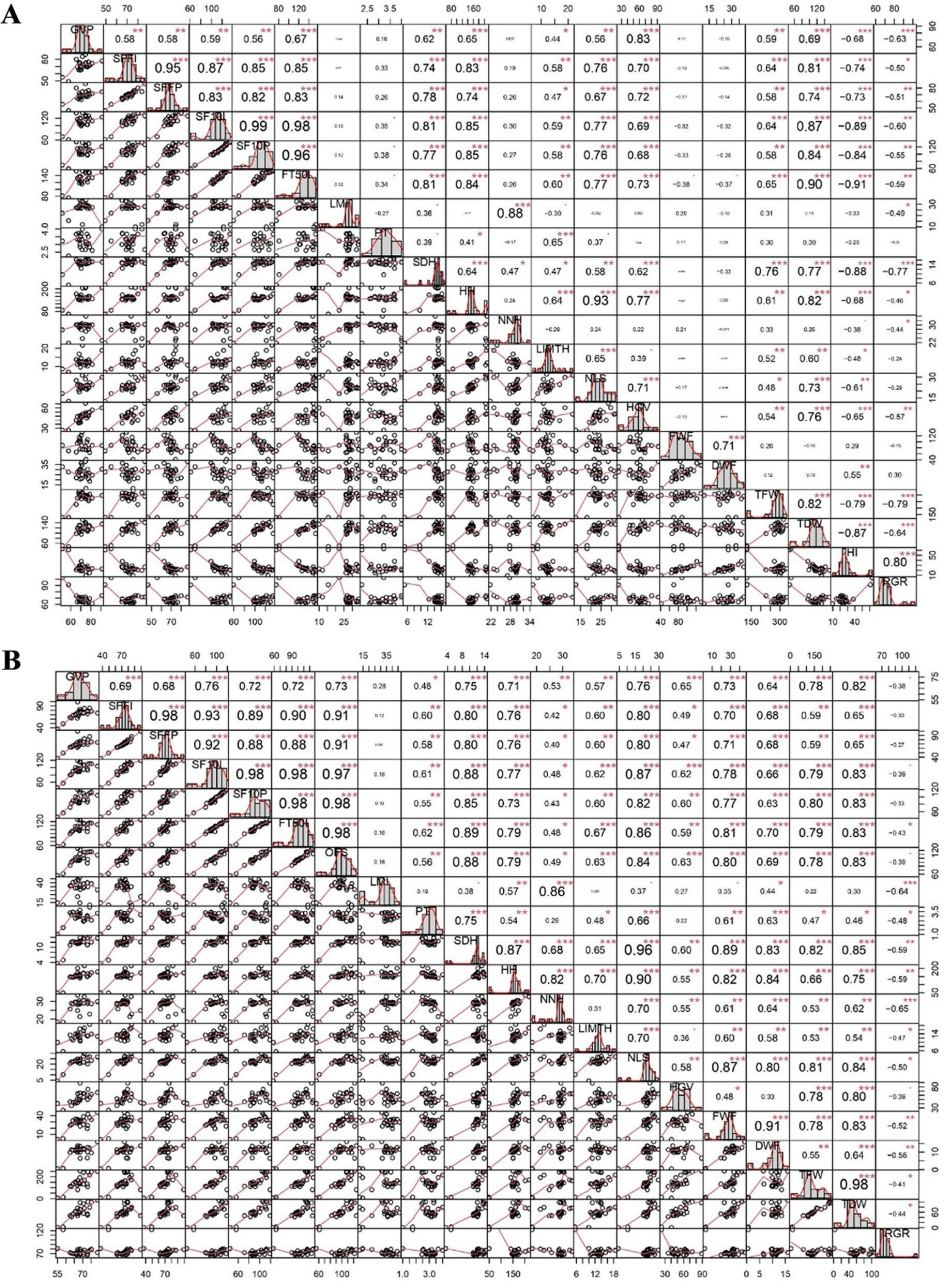
Correlation coefficient between 14 morphological traits and 6 female and 7 male phenological traits for 25 native cannabis populations of Iran in female plants (**A**) and male plants (**B**). Significance of correlation is indicated with *, **, and *** corresponding to *P* ≤ 0.05, 0.01, and 0.001, respectively. Abbreviations; LMI: Length of Main Inflorescence, PT: Plant Type (1 to 4). SDH: Stem Diameter on Harvest day, HH: Height on Harvest day, NNH: Number of Nodes on the main stem on Harvest day, LIMTH: Length of Internode in the Middle Third of the main stem on Harvest day, NLS: Number of Lateral Shoot, HGV: Height to GV Point, FWF: Fresh Weight of Flowers, DWF: Dry Weight of Flowers, TFW: Total Fresh Weight, TDW: Total Dry Weight, HI: Harvest Index, RGR: Relative Growth Rate, GVP: GV Point, SFFI: Start Flower Formation Time in Individuals, SFFP: Start Flower Formation Time in 50% Population, SF10I: Start 10% Flowering Time in Individuals (10% of bracts formed), SF10P: Start 10% Flowering Time in 50% Population (10% of bracts formed), FT50I: Flowering Time 50% in Individuals (50% of bracts formed), OFS: First Opened Staminate Flowers

**Fig. 8:**
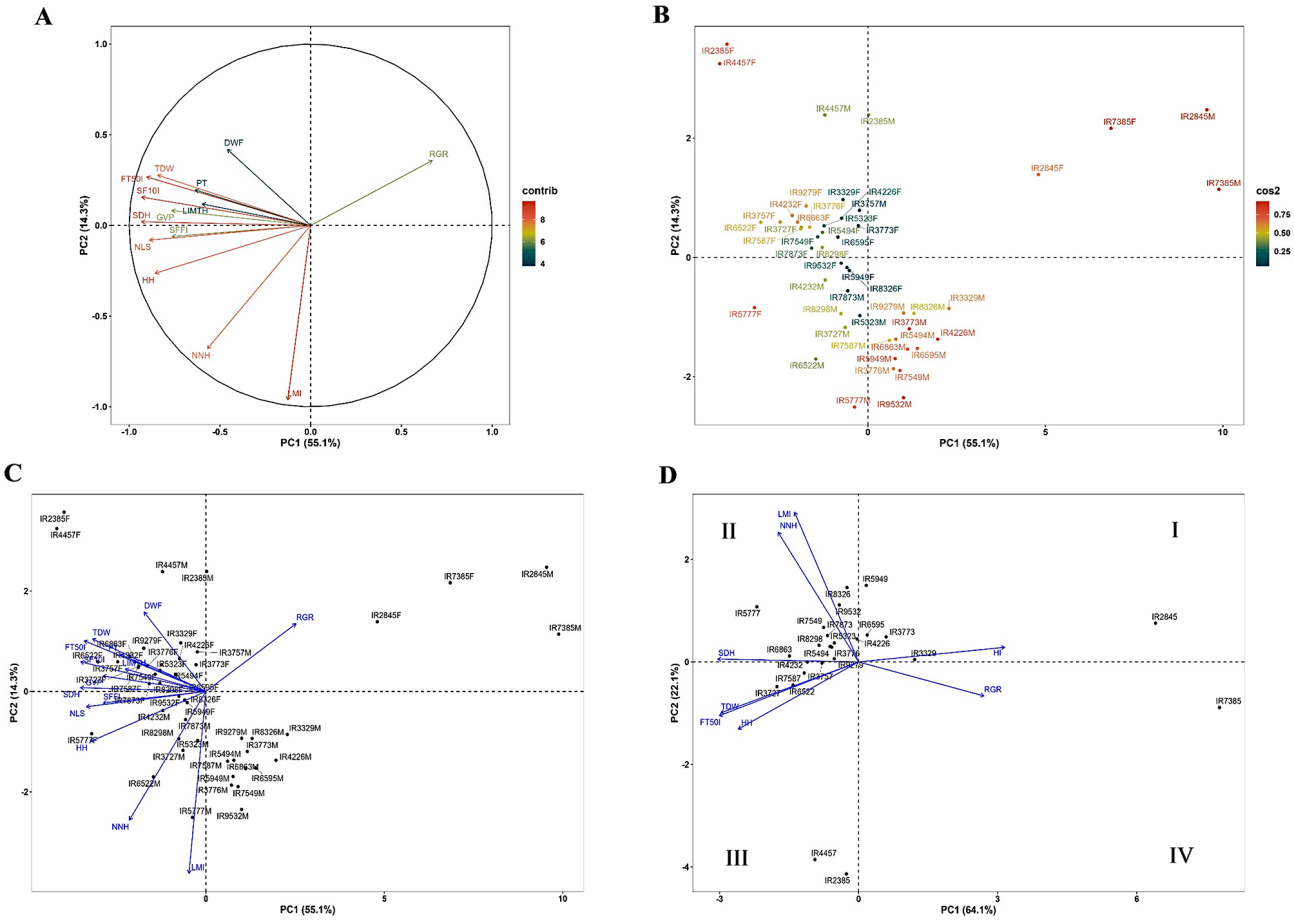
Principal component analysis (PCA) for 25 native cannabis populations in Iran. (**A)** Illustration of the relationship between 14 morphological and phenological traits. The contribution of each trait to the principal component is visualized using a specific color, which is represented by a color bar alongside the plot. (**B**) Indicating the extent of influence of each population (female and male) on the formation of two principal components. (**C**) Illustration of the distribution of populations (female and male) in four parts and highlights the role and importance of variables (vectors) in distinguishing populations and different data patterns. (**D**) Illustrating the relationship between eight traits with high breeding values and female plants distribution. Abbreviations; GVP: GV Point, SFFI: Start Flower Formation Time in Individuals, SF10I: Start 10% Flowering Time in Individuals (10% of bracts formed), FT50I: Flowering Time 50% in Individuals (50% of bracts formed), LMI: Length of Main Inflorescence, PT: Plant Type (1 to 4), SDH: Stem Diameter on Harvest day, HH: Height on Harvest day, NNH: Number of Nodes on the main stem on Harvest day, LIMTH: Length of Internode in the Middle Third of the main stem on Harvest day, NLS: Number of Lateral Shoot, DWF: Dry Weight of Flowers, TDW: Total Dry Weight, RGR: Relative Growth Rate, F: Female and M: Male

To explore the relationships between morphological and phenological traits, PCA and correlation matrix were used (Figs. 7 and 8). As shown in Fig. 7A, it is evident that various phenological traits (GVP, SFFI, SFFP, SF10I, SF10P, and FT50I) exhibited significant correlations with nine morphological parameters (SDH, HH, LIMTH, NLS, HGV, TFW, TDW, HI, and RGR). Notably, HI and RGR displayed negative correlations, while the relations with the rest of the parameters were positive. However, no significant correlations were found between phenological traits and parameters including LMI, PT, NNH, FWF, and DWF. RGR, in particular, demonstrated significant correlations with various parameters, including a strong positive correlation with HI (p $\le$ 0.001, *r* = 0.80) (Fig. 7A). Female plants exhibited the highest correlation value in HH and NLS (p $\le$ 0.001, *r* = 0.93). The DWF parameter showed significant correlations exclusively with FWF and HI, with correlation coefficients of p $\le$ 0.001, *r* = 0.71 and p $\le$ 0.001, *r* = 0.55, respectively. TDW and TFW parameters were found to have similar relations with the other morphological traits, showing significant negative relations with RGR and HI, along with significant correlations with parameters including SDH, HH, LIMTH, NLS, and HGV. Finally, a strong positive relation was found between LMI and NNH (p $\le$ 0.001, *r* = 0.88).

The correlation analysis for male plants showed stronger relationships between the examined traits (Fig. 7B). In addition, the correlation patterns for male plants were significantly different from those of female plants. For male plants, various phenological traits (GVP, SFFI, SFFP, SF10I, SF10P, and OFS) showed significant and positive correlations with all morphological traits, except for LMI and RGR, with which only FT50I exhibited a significant correlation (p $\le$ 0.001, *r* = -0.43). RGR was found to have a negative relation with all positively correlated traits and showed the highest correlation coefficient with NNH (p $\le$ 0.001, *r* = 0.65) and LMI (p $\le$ 0.001, *r* = 0.64) (Fig. 7B). There was also a high correlation coefficient between SDH and NLS (p $\le$ 0.001, *r* = 0.96). Parameters including FWF and DWF, related to the weights of flowers, demonstrated the highest correlation values with morphological traits, including SDH, HH, and NLS. Moreover, TFW and TDW had no significant correlations with the LMI trait and showed a negative relation with RGR.

To minimize the effects of highly overlapping traits, PCA was conducted on a total of fourteen traits including both male and female plants (Fig. 8). The results of PCA analysis showed that three output factors involved eigenvalues of above one, specifically λ_1_ = 7.71 and λ_2_ = 2 for the first and second factors, respectively. These first two factors collectively accounted for 69.44% of phenotypic variation, consisting of 55.1% and 14.3% attributed to the first and second factors, respectively. In the biplot, variables were loaded onto the first two components, showcasing the contribution value, direction, and strength of each trait. The highest contribution in PC2 was associated with LMI and NNH, while the rest of the traits contributed to PC1 (Fig. 8A). RGR showed the highest dissociation among all traits. Positive correlations were observed between phenological traits including FT50I, SF10I, SDH, and HH, and the functional trait TDW, while DWF, PT, and LIMTH had the lowest contribution.

Several populations displayed the highest contributions in the first two factors, such as IR7385 and IR2845 in both male and female plants, as well as IR2385, IR4457, and IR5777 in female plants and IR5949, IR9532, IR3773, and IR4226 in male plants (Fig. 8B). These two factors effectively separated male and female plants, except for five populations including IR7385, IR2845, IR2385, IR4457, and IR3757, where both male and female plants were found to be in the same zone (Fig. 8C). The results of PCA conducted on female plants for eight traits with high breeding values showed that two output factors with eigenvalues of greater than one (λ_1_ = 5.12 and λ_2_ = 1.76) (Fig. 8D). In total, these two factors accounted for 86.19% (PC1 = 64.1% and PC2 = 22.1%) of the phenotypic variation. A clear separation was observed between HI and RGR traits, compared to others. Populations in the first zone (I) had the highest HI values, while populations in the second zone (II) had the highest LMI and NNH (PC2) values. In addition, the highest values of TDW, FT50I, and HH were observed in the third zone (III), and the highest RGR value was found in the fourth zone (IV), which only included the IR7385 population (Fig. 8D).

## Discussion

Despite significant progress in cannabis research, our understanding of the genetic makeup and population structure remains somewhat limited. Moreover, regulations established by international drug control organizations, over past decades, have imposed restrictions on research into various aspects of cannabis genetic resources [2, 25]. Consequently, the untapped potential within the genetic reservoir of cannabis has been underutilized regarding the development of new cultivars. Nevertheless, gaining awareness of phenotypic features, particularly those closely tied to genetic structures, can accelerate the selection process and its overall efficiency for breeders [26]. In this study, we have performed an extensive evaluation of native cannabis populations, often referred to as landraces, within Iran, one of the original gene pools of cannabis [20].

Considering the importance of this subject, phenological parameters were initially examined methodically, based on the descriptor of Mediavillia from the time of seed cultivation [24]. The entire population progressed for 43 days until the appearance of the fifth node; however, following the 43rd day, differences in various stages were observed among populations, and between male and female plants in each population. Given the findings of a previous study, phenological development in cannabis is linked to its geographical origin or, in other words, the latitude to which it has adapted [27]. This suggests that the present diversity observed in traits, populations, and individuals within a population may be influenced by genetic factors, environmental forces, and their interactions [28]. As a result, an understanding of the variance within these examined traits in populations that have been adapted to an environment in a long period of time can provide valuable insights into the mechanisms underlying the formation of particular phenotypes. This knowledge also aids in investigating the general sustainability or plasticity of a trait or cultivar.

In another study, four commercial fibrous cultivars of early, medium, and late-flowering types were cultivated at various times and their performances were assessed and utilized to develop models for estimating flowering times [29]. The diversity in flowering times has multiple dimensions, often rooted in the intended use of the cultivated cultivar and the specific environment in which it is grown. For instance, in outdoor cultivation, factors such as latitude, weather conditions, day length, and growth duration can significantly impact flowering times. This is essential knowledge considering the broad range of applications for cannabis in industries, pharmacology, and medicine [30, 31]. For instance, in the context of seed production, early-flowering and late-flowering cultivars are recommended for high-latitude regions (short growth season) and low-latitude areas (long growth season), respectively [32]. Late-flowering cultivars are mostly used in the northern regions with high latitudes for fiber production [32]. Understanding the correlation between flowering times and the desired end product is pivotal in cannabis cultivation and is directly related to the cultivar’s adaptation to specific environmental conditions and growth goals.

Cannabis is a short-day plant, and its flowering process is highly influenced by day length [27, 29, 33, 34]. The findings showed that cannabis can be classified into three genetic lineages associated with different latitudes: high latitude (40$^\circ$ North), medium latitude (30$^\circ$-40$^\circ$ North), and low latitude (30$^\circ$ North) [35]. Each group has gradually adapted to the climate conditions of its respective latitude. For instance, the high latitude group displays features like short plant stature, thin stem, fewer branches, and short growth period, whereas the low latitude group exhibits precisely the opposite traits [35]. Iran, geographically located between 25-40$^\circ$ North latitude [36]l three of these latitudinal categories. This geographical diversity, in conjunction with the observed phenotypic diversity and significant differences in growth periods in indigenous Iranian cannabis populations, provides a plausible explanation for the classification into three distinct three groups based on their time of flowering [35, 37]. It is important to note that the genetic control of flowering in cannabis is controlled by several key genes, and despite the limited research in this area, the initial natural diversity of the plant remains largely unknown. Exploring this natural diversity becomes instrumental in the development of cultivars with specific flowering behaviors [38].

The examinations of morphological traits showed significant diversity across the analyzed parameters. Statistical indicators such as coefficient of variation (CV), standard deviation, and range clearly demonstrated the extent of variations in each trait, for both male and female plants. A study conducted by other researchers explored various morphological traits, including stem diameter, height, internode length, the number of nodes, the dry weight of the flower, and harvest index, in 121 genotypes with distinct cannabinoid profiles [22]. Another investigation into diversity among 123 accessions revealed significant variations in traits like stem diameter, dry weight of the flower and stem, and plant height [39]. Their findings highlighted the high diversity within morphological traits. Considering the highly heterozygous nature of the cannabis genome, which gives rise to unique properties in each individual, it is reasonable to expect variation in results across different traits, in distinct environments, and with different genotypes. Moreover, most similar studies carried out have been conducted using commercial genotypes, which have experienced a reduction in diversity due to consecutive selections over time. Therefore, the results of this study demonstrate differences in minimum-maximum values for each trait compared to similar research. One of the main drivers of diversity in cannabis is its dioecious nature [39, 40], where male plants complete their vegetative phase and transition into the reproductive phase more swiftly than female plants. This difference in flowering times between male and female plants contributes to the diversity and increased heterozygosity observed in cannabis [41]. The application of morphological parameters has become a standard method in assessing diversity, particularly in plants characterized by variable and diverse characteristics [42].The extent of broad-sense heritability (H^2^) of both phenological and morphological traits offers insights into the degree of variance in phenotypes (VP) that can be attributed to genetic variance (VG) [43, 44]. Researchers provided three interpretations for this parameter [45]: (I) it represents a regression coefficient between unobservable genotypic value and observable phenotypic value; (II) it measures the correlation between genotypic value and the anticipated phenotypic value; and (III) it corresponds to selection and its impact, defined as the ratio of the selection differential (S; the mean difference of a trait in a population prior to selection) to the response differential (R; the change in the mean value of a trait in a population from one generation to the next resulting from natural or artificial selection) [45]. When comparing male and female plants, it can be concluded that there was a significant stability among parameters from the appearance of solitary flowers up to FT50I. However, among female plants, H^2^ exhibited a variable declining trend from the appearance of solitary flowers to the final flowering stages (FT50I). This aligns with the findings of researchers who examined 123 accessions and estimated H^2^ in the early-stage flowering and complete flowering at 0.95 and 0.94, respectively [39]. However, the estimates were not gender specific. Additionally, H^2^ estimates in 14 different morphological parameters demonstrated significant differences between male and female plants, with the most notable difference was found between DWF and FWF. Previous studies have reported similar H^2^ values for parameters related to inflorescence weight in female plants (0.33). However, to the best of our knowledge there are no reports for male plants [22, 23, 46, 47]. Given the predominantly high H^2^ value in most traits, it can be concluded that a significant part of this diversity possibly stems from genetic influences. Furthermore, the results indicate that selection in male plants can be effectively bring about changes in traits with high heritability across subsequent generations. Traits with low H^2^ are less suitable for reproducing and transferring genetics to the next generation, and they tend to have a high phenotypic diversity in various environments. On the other hand, traits with high H^2^ are mostly controlled by genetic effects making them particularly relevant in plant breeding [48, 49].

Populations were clustered based on phenological and morphological traits using Heatmap. Morphological, phenological, and agronomic traits are mostly utilized for an initial detection of germplasm and are of particular importance as basic information for breeders when examining genetic diversity [6, 50]. Therefore, selecting and using traits to distinguish between plants for breeding can be considerably useful and effective [51].

The results of PCA, clustering and correlation were highly overlapping; however, using each set of results contributed to the analysis of the results in this study from different perspectives. While some correlations aligned with prior findings, such as the relationship between DWF HH and SDH, other correlations, like HH and HI, differed from earlier studies [22, 39]. Interestingly, a positive connection between phenological traits and morphological characteristics was observed in our results, in contrast to previous research [22]. The number of nodes and their spacing significantly influenced plant height. Additionally, Days to Maturation (DTM) emerged as a prominent differentiator of genotypes in previous research [6], whereas in our study, RGR and HI played a similar role in distinguishing between traits. This multifaceted approach to analyzing the results offers a comprehensive understanding of the cannabis plant’s traits and their correlations. The existence of high correlations among different traits facilitates the indirect selection of important traits. As a result, traits with high correlations can be used for population selection and trait modification. Specifically in terms of functional parameters, factors such as environmental impact, polygene, low heritability, linkage, epistasis, and non-additive genes across functional traits have reduced the efficiency of early-generation selection (EGS) in this initial generations. Therefore, using the indirect breeding method and employing traits with high correlation and high heritability can be useful for selection [48, 49, 52, 53].

## Conclusion

Phenotyping plays a vital role in plant breeding. It serves as a means to identify important features in plants, allowing for the selection of superior genotypes and thereby improving plant breeding practices. Phenotyping of indigenous populations is a cornerstone in studies aiming to develop plants with enhanced performance, resilience against environmental stresses, increased genetic diversity, and other desirable characteristics. This study has unveiled significant phenotypic diversity within and among populations, offering a comprehensive outlook for future breeding initiatives by exploring a wide range of morphological and phenological traits. The genetic diversity within indigenous cannabis populations harbors potential for diverse applications. For instance, early-flowering populations characterized by high RGR, HI, and shorter stature are well-suited for breeding programs targeting cultivar development in vertical farming systems. Given the high level of heritability for most of the traits, future molecular studies can also be employed to investigate the genetic basis of these traits to develop molecular markers and accelerate breeding efforts to develop varieties with medicinal importance.

## Methods

### Plant materials

Cannabis seeds were sourced from different regions in Iran to ensure genetic diversity and regional adaptability. Utilizing the Köppen-Geiger climate classification method [54–56], with five climatic zones and latitudes between 25° and 40° North and Longitudes 45° and 65° East (Fig S2). The seeds were collected from traditional farmers and local markets and organized into groups according to their respective regions of origin, with each population representing a unique geographical area. In total, 25 distinct indigenous populations were collected for this study. The geographical information system (GIS) and ArcGIS Online (Developed by Esri) were used to interpolate seed collection locations and create a map to illustrate their spatial distribution across different climatic regions [55].

Before starting the experiment, an initial germination test was conducted to assess key parameters, including the Final Germination Percentage (FGP), Germination Rate (GR) and Mean Germination Time (MGT) of these seeds. Additional details are available in Fig S1. The germination test was performed based on a completely randomized design (CRD) with 3 replications and 25 observations. The experiment was carried out in the germinator at the laboratories of the Department of Horticultural Sciences at Ferdowsi University of Mashhad, Iran. The germinator maintained a 16 h light and 8 h darkness, with temperatures set at a constant 22 °C, and a relative humidity of approximately 70%. The germination rate was assessed 7 days after the initiation of the experiment.

### Experimental design and growing conditions

Cannabis seeds, 20 per population, were sown in multicellular trays with a mixture of cocopeat, peat moss, vermicompost, and perlite with a ratio of 2:1:1:1 and kept for 30 days. The cultivation environment was maintained with an average relative humidity of 55 ± 15%, with a temperature of 27 ± 1 °C during the day and 20 ± 1 °C at night, seedlings were exposed to natural sunlight. Then, 375 seedlings were transplanted into growth bags with soil substrate in the greenhouse complex of the Ferdowsi University of Mashhad, Iran (36^°^16ʹN and 59^°^36ʹE with an altitude of 985 m), using a randomized complete blocks design (RCBD) with three block and five observations. The plant cultivation substrate was a hand-made mixture consisting of garden soil, leaf mold, sand, and pearlite with a proportion of 3:1:1:1 (see Table S3). The plants were grown in two separate 60 m^2^ units with a temperature set to 27 ± 1 °C during the day and 20 ± 1 °C at night, with an average relative humidity of 55 ± 15%, Throughout the entire growth period, plants were exposed to natural sunlight. Cultivation time was adjusted based on day-length data in the region. To ensure completion of the vegetative growth phase before entering the reproductive phase, the switch-off period saw an increase in day length from 13.17 h at the start to 14.29 h on the 60th day. The plants were irrigated three times a week. One of these irrigation sessions was adjusted to maintain a pH level between 5.5 and 6. Additionally, based on the soil analysis results, the plants received weekly fertigation with a nutrient solution consisting of NPK ratio of 20-12-20 with 20 Ca (753 ppm) and 20-25-25 with 16 Ca (858 ppm) during the vegetative and reproductive stage, respectively, up to the fourth week after the onset of flowering (Details of the fertilizer mix can be found in Table S4). To allow the plants to express their natural phenotype, no physical alterations such as pruning or topping were performed. However, during the growth period, any dead, damaged, or yellowing leaves were removed. Using Spatial Analysis (SA), the resulting unit was divided into 25 rows and 15 columns, and each block entailed 5 columns. Subsequently, different populations were randomly placed in different rows in each block. Additionally, blocks were separated via corridors with a width of 50 cm. In general, 16 pots were placed within each square meter of the unit. Accordingly, to prevent pollination at the onset of the reproductive phase, female plants were separated from male plants and transferred to another unit. As a result, this number was reduced to eight pots in one m^2^.

### Examined traits

#### Recording the descriptor events of phenological stages

**Fig. 9:**
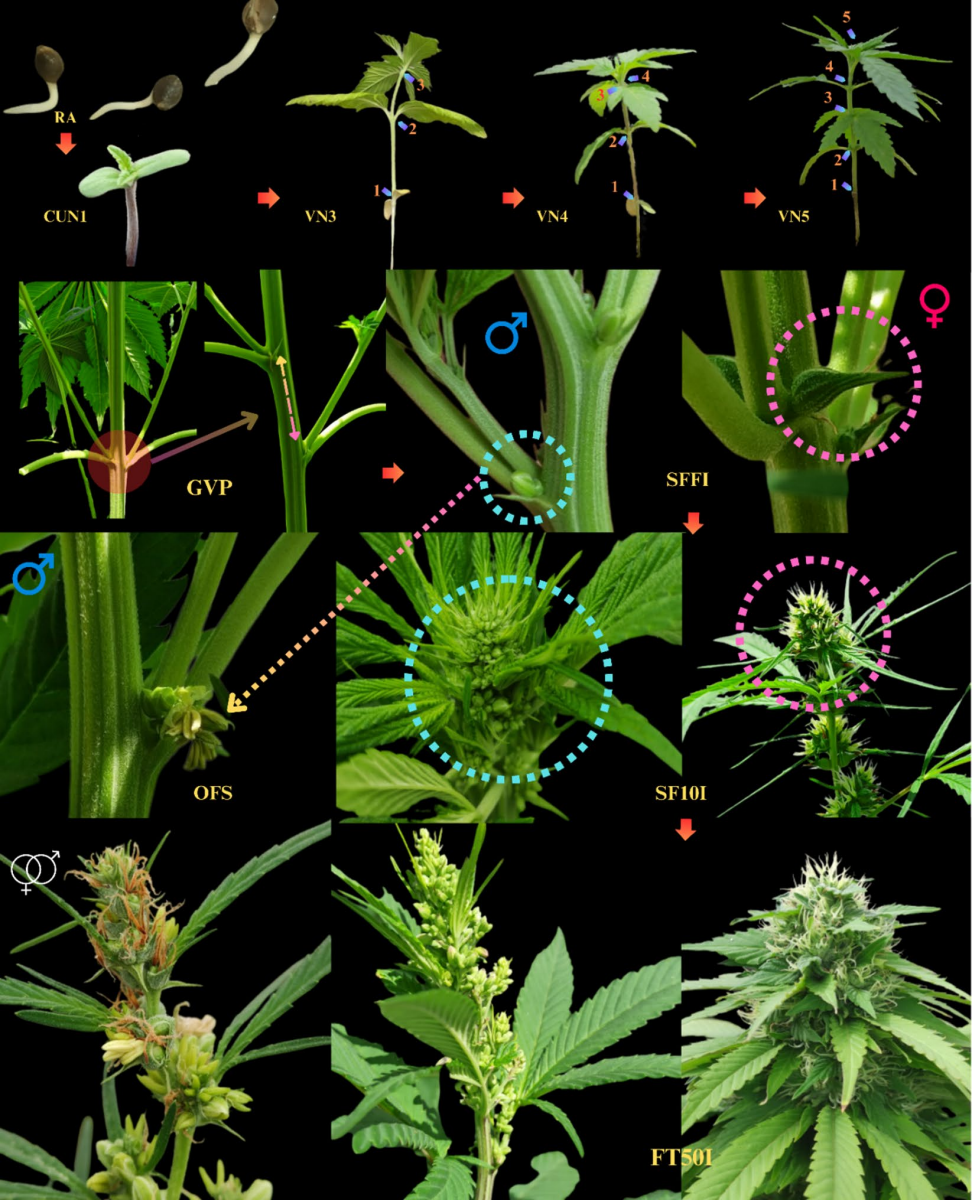
Visual descriptor depicting different phenological stages in male and female plants. RA: Radicle Apparent, CUN1: Cotyledons Unfolded (1st node), VN3: Vegetative Stage (2nd leaf pair), VN4: Vegetative Stage (3rd leaf pair), VN5: Vegetative Stage (4th leaf pair), GVP: GV Point, SFFI: Start Flower Formation Time in Individuals, SF10I: Start 10% Flowering Time in Individuals (10% of bracts formed), FT50I: Flowering Time 50% in Individuals (50% of bracts formed), OFS: First Opened Staminate Flowers

All events in stages including seed germination, vegetative, and reproductive were recorded according to the number of days since cultivation, based on the descriptor proposed by Mediavillia et al., (1998) (Table 4; Fig. 9). Notably, certain changes were made to this descriptor [24].

**Table 4:** The recorded descriptor events of phenological stages in male and female cannabis plants

Growth and Development Stages		Abbreviation	Definition
Germination and Emergence	Radicle Apparent	RA	Based on the germination test, where the radicle appeared in 50% of seeds.
	Cotyledons Unfolded	CUN1	Assessed during seedling transplantation, where 50% of cotyledons (1st node) in the entire population unfolded.
Vegetative Stage	Third node	VN3	Presence of four leaves, each with three leaflets.
	Fourth node	VN4	Presence of six leaves, each with five leaflets.
	Fifth node	VN5	Presence of eight leaves, each with seven leaflets.
Reproductive Stage	GV Point	GVP	This is the moment when the phyllotaxis of buds’ transitions from the opposite state to the alternate state. The criterion for this transition is a minimum distance of 0.5 cm between petioles of alternate leaves. This stage marks the entry of the plant into its reproductive phase.
	Start Flower Formation Time in Individuals	SFFI	The appearance of the first bud in the angle between primary and secondary branches which is in the form of a bell-shaped solitary flower or closed sepals in the male plant; in the female plant, it is in the form of a symmetrical calyx where two styles appear that are commonly white and has a hairy shape. The criterion was the appearance of the first flower in individuals constituting a population in each experimental block.
	Start Flower Formation Time in 50% Population	SFFP	Characterized by the appearance of the first flower in half of the individuals within a population in each experimental block.
	Start 10% Flowering Time in Individuals	SF10I	Characterized by the appearance of a minimum of 10% of the main inflorescence (tip of the main stem) in individuals within a population in each experimental block. The inflorescence of male plants is formed by the accumulation of flower in the upper part of the main stem (rachis) and the formation of a few leaves. Conversely, in female plants, inflorescence is developed on the tip of the main stem and appears in the form of raceme inflorescence.
	Start 10% Flowering Time in 50% Population	SF10P	Characterized by the appearance of 10% of inflorescence in half of the individuals within a population in each experimental block.
	Flowering Time 50% in Individuals	FT50I	Characterized by the appearance of 50% of the main inflorescence in individuals within a population in each experimental block.
	First Opened Staminate Flowers	OFS	This stage is marked by the opening of the sepals of the first bell-shaped solitary flower in the male plant. During this stage, mature yellow pollen is dispersed into the environment after the anther sac is torn apart. Characterized by the opening of the first anther in the first individual within a population in each experimental block.

#### Morphological traits

**Fig. 10:**
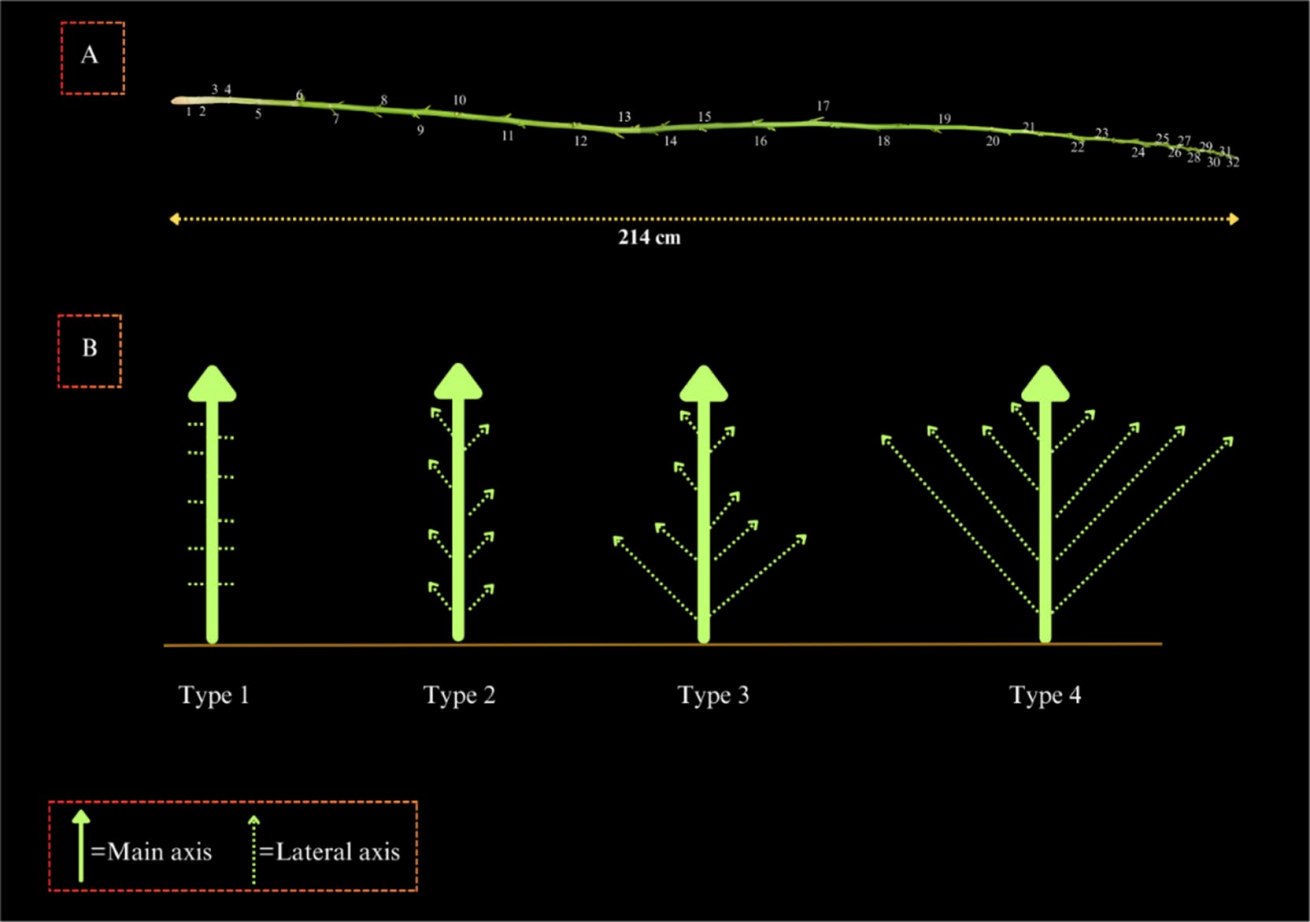
Schematic illustration of plant morphology and node arrangement; (**A**) all leaves and branches were intentionally removed to facilitate the node counting along the main axis. Even after the phyllotaxy transitioned from opposite to alternating, each pair of nodes was considered as a single node. (**B**) four distinct plant morphotypes (PT 1–4)

Morphological traits including the Length of Main Inflorescence (LMI), Plant Height (HH), the Length of Internode in the Middle Third of the main stem (LIMTH), and Height to GV Point (HGV) were measured using a tape measure with 0.01 m precision, at the end of the cultivation period and in the time of harvesting male and female plants. In addition, the Number of Nodes on the main stem (NNH) (Fig. 10A) and Number of Lateral Shoot (NLS) were counted. Stem diameter (SDH) at 5 cm above soil surface was measured for all individuals, using calipers with an accuracy of 0.01 mm. The Fresh and Dry Weights of the Flower (FWF and DWF) and the total weight of the aerial organ were measured as well (Total Fresh Weight (TFW) and Total Dry Weight (TDW)). To this aim, a digital scale (AND-GF3000) with an accuracy of 0.001 g was used to separately measure the fresh weights of the entire parts. Subsequently, to measure the dry weights, samples were maintained at room temperature (25$?$) for 15 days and then weighed again using the same digital scale. Then, as illustrated in Fig. 10B, plants were classified into four distinct plant types (PT 1–4) based on their growth characteristics. Type 1 plants featured lateral branches attached to the main axis, while lateral branches were not visible. Type 2 plants displayed lateral branches positioned at a uniform distance from the main axis, maintaining consistent length from base to tip. In Type 3 plants, lower lateral branches were longer than upper branches. Type 4 plants exhibited lower branches of equal length to the main axis, with all branches converging at the same level along the main axis.

Finally, we calculated the Harvest Index (HI) for the yield of female buds which can be utilized as a commercial index through the following Eq. (1):

1 $$\varvec{H}\varvec{I}\left(\varvec{\%}\right)=\left(\frac{\varvec{E}\varvec{c}\varvec{o}\varvec{n}\varvec{o}\varvec{m}\varvec{i}\varvec{c} \,\varvec{Y}\varvec{i}\varvec{e}\varvec{l}\varvec{d}}{\varvec{T}\varvec{o}\varvec{t}\varvec{a}\varvec{l} \,\varvec{B}\varvec{i}\varvec{o}\varvec{l}\varvec{o}\varvec{g}\varvec{i}\varvec{c}\varvec{a}\varvec{l} \,\varvec{Y}\varvec{i}\varvec{e}\varvec{l}\varvec{d}}\right)\times 100$$

In this equation, *Economic Yield* is defined as the Dry Weight of Flowers (DWF) and *Total Biological Yield* is defined as the Total Dry Weight (TDW) [22].

The relative growth rate (RGR) was also calculated using following Eq. (2) and according to g.g^− 1^.day^− 1^:

2 $$\varvec{R}\varvec{G}\varvec{R}=\frac{{\varvec{ln}\,\varvec{T}\varvec{D}\varvec{W}}_{2}-{\varvec{ln}\,\varvec{T}\varvec{D}\varvec{W}}_{1}}{{\varvec{t}}_{2}-{\varvec{t}}_{1}}$$

In this equation, the biomass increases from TDW_1_ to TDW_2_, on average during a time interval of t_1_-t_2_. *TDW*_*2*_ and *TDW*_*1*_ are the dry weights of plants at the time of harvest and in the seedling transplantation stage, respectively. Consequently, *t*_*2*_ and *t*_*1*_ represent the time of harvest and seedling transfer, respectively. *1n* signifies the Natural Logarithm [57].

### Statistical analysis

Data were initially assessed using the Outlier Grubbs technique [58], to identify and remove outliers. Then, the skewness and kurtosis analysis [59], were performed using Minitab® Statistical Software 21 [60], to examine the normality of data and residual errors based on the type of distribution. The missing data were imputed using the classification and regression tree (CART) method implemented in the AllInOne package [61] in R software version 4.3.1. Spatial Analysis (AR1⊗AR1) was also utilized to correct the possible spatial effects and reduce environmental impact between 15 and 25 experimental columns and rows, respectively. Data were fitted using the same approach, with population and block serving as fixed and random factors, respectively. The performance of the model was evaluated using four parameters including Root Mean Square Error (RMSE; Eq. 3), Mean Squared Error (MSE; Eq. 4), Normalized Root Mean Squared Error (NRMSE; Eq. 5), and Coefficient of Variance (CV; Eq. 6). Additional details are available in Table S5 [61–63].

3 $$\text{RMSE}\text{ =}\sqrt{\frac{1}{\varvec{n}}\sum _{\varvec{i}=1}^{\varvec{n}}({\varvec{y}}_{\varvec{i}}^{\varvec{{\prime }}}-{{\varvec{y}}_{\varvec{i}})}^{2}}$$

4 $$\varvec{M}\varvec{S}\varvec{E}= \frac{1}{\varvec{n}}\sum _{\varvec{i}=1}^{\varvec{n}}({\varvec{y}}_{\varvec{i}}^{\varvec{{\prime }}}-{{\varvec{y}}_{\varvec{i}})}^{2}$$

5 $$\varvec{N}\varvec{R}\varvec{M}\varvec{S}\varvec{E}=\frac{1}{\stackrel{-}{\varvec{y}}}\sqrt{\frac{1}{\varvec{n}}\sum _{\varvec{i}=1}^{\varvec{n}}({\varvec{y}}_{\varvec{i}}^{\varvec{{\prime }}}-{{\varvec{y}}_{\varvec{i}})}^{2}}$$

6 $$\varvec{C}\varvec{V} \left(\varvec{\%}\right)=\frac{1}{\stackrel{-}{\varvec{y}}}\sqrt{\frac{1}{\varvec{n}}\sum _{\varvec{i}=1}^{\varvec{n}}{{(\varvec{y}}_{\varvec{i}}-\stackrel{-}{\varvec{y}})}^{2}}\times 100$$

where:

n = The number of observations.

${y}_{i}^{{\prime }}$ = The predicted value in observation *i*.

${y}_{i}$ = The actual value in observation *i*.

$\stackrel{-}{y}$ = The mean of the data values

Broad-sense heritability (H^2^) was calculated in the AllInOne package [61] using following Eq. (7):

7 $${\varvec{H}}^{2}=\frac{{\varvec{\sigma }}_{\varvec{G}}^{2}}{{\varvec{\sigma }}_{\varvec{G}}^{2}+{\varvec{\sigma }}_{\varvec{G}\times \varvec{E}}^{2}+\frac{{\varvec{\sigma }}_{\varvec{?}}^{2}}{\varvec{n}.\varvec{B}}}$$

where:

${\sigma }_{G}$= The genetic variance.

${\sigma }_{G\times E}$ = The variance due to genotype-by-environment interactions.

${\sigma }_{\epsilon}$ = The residual variance.

$n.B$ = The number of blocks.

The studied populations and traits were clustered based on between-group linkage, using the Pheatmap package [64] in R. To this end, data was standardized using the Z score (Eq. 8).

8 $$\varvec{Z}= \frac{\varvec{X}-\varvec{\mu }}{\varvec{\sigma }}$$

where:

X = The single raw data value.

µ = The mean.

σ = The standard deviation.

Principal Component Analysis (PCA) was conducted using the Factoextra package [65] in R software version 4.3.1. Moreover, packages including Performance Analytics [66] and corrplot [67] in R as well as the Pearson method were utilized to examine the correlation between traits. The significance of the traits was determined through variance analysis using Minitab® Statistical Software 21 [60]. Furthermore, the Least Significant Differences (LSD) test at 1% significance level was used to examine mean comparison.

### Electronic supplementary material

Below is the link to the electronic supplementary material.

Supplementary Material 1

Supplementary Material 2

Supplementary Material 3

Supplementary Material 4

Supplementary Material 5

Supplementary Material 6

Supplementary Material 7

## Data Availability

The raw datasets obtained during the current study are available from the corresponding author on reasonable request.

## Abbreviations

LMI: Length of Main Inflorescence
PT: Plant Type (1 to 4)
SDH: Stem Diameter on Harvest day
HH: Height on Harvest day
NNH: Number of Nodes on the main stem on Harvest day
LIMTH: Length of Internode in the Middle Third of the main stem on Harvest day
NLS: Number of Lateral Shoot
HGV: Height to GV Point
FWF: Fresh Weight of Flowers
DWF: Dry Weight of Flowers
TFW: Total Fresh Weight
TDW: Total Dry Weight
HI: Harvest Index
RGR: Relative Growth Rate
RA: Radicle Apparent
CUN1: Cotyledons Unfolded (1st node)
VN3: Vegetative Stage (2nd leaf pair)
VN4: Vegetative Stage (3rd leaf pair)
VN5: Vegetative Stage (4th leaf pair)
GVP: GV Point
SFFI: Start Flower Formation Time in Individuals
SFFP: Start Flower Formation Time in 50% Population
SF10I: Start 10% Flowering Time in Individuals (10% of bracts formed)
SF10P: Start 10% Flowering Time in 50% Population (10% of bracts formed)
FT50I: Flowering Time 50% in Individuals (50% of bracts formed)
OFS: First Opened Staminate Flowers
