# Supplementary material for: Characterization of indigenous populations of cannabis in Iran: a morphological and phenological study
Source: BMC Plant Biol. 2024 Feb 29;24:151. doi: 10.1186/s12870-024-04841-y (PMC10902964; doi:10.1186/s12870-024-04841-y)
Supplement: Supplementary file 1 — Supplementary Material 1 [file 12870_2024_4841_MOESM1_ESM.docx]

**Characterization** **of Indigenous Populations of Cannabis in Iran: A Morphological and Phenological Study**

Mehdi Babaei^1, 2, 3, 4, 5^, Hossein Nemati^1, *^, Hossein Arouiee^1^, Davoud Torkamaneh ^2, 3, 4, 5^

^1^ Department of Horticultural Sciences, Ferdowsi University of Mashhad, Azadi Square, Mashhad, 9177948974, Razavi Khorasan, Iran.

^2^ Département de Phytologie, Université Laval, Rue de l'Université, Québec City, G1V 0A6, Québec, Canada.

^3^ Institut de Biologie Intégrative et des Systèmes (IBIS), Université Laval, Rue de l'Université, Québec City, G1V 0A6, Québec, Canada.

^4^ Centre de recherche et d’innovation sur les végétaux (CRIV), Université Laval, Rue de l'Agriculture Québec City, G1V 0A6, Québec, Canada.

^5^ Institute Intelligence and Data (IID), Université Laval, Rue de l'Agriculture Québec City, G1V 0A6, Québec, Canada.

*Corresponding author. E-mail: [nemati@um.ac.ir](mailto:nemati@um.ac.ir)

Contributing authors : [mehdi.babaei.1@ulaval.ca](mailto:mehdi.babaei.1@ulaval.ca); [aroiee@um.ac.ir](mailto:aroiee@um.ac.ir); [davoud.torkamaneh.1@ulaval.ca](mailto:davoud.torkamaneh.1@ulaval.ca)

**Assessing the germination performance of 25 indigenous cannabis populations**

The seed germination experiment for 25 indigenous populations from Iran was assessed according to the International Seed Testing Association (ISTA) standards in a completely randomized design (CRD) with 3 replications and 25 observations. Each Petri dish (9 cm) contained 25 seeds, and the germination process took place in a germinator for 7 days under controlled conditions (22 °C, 70% humidity, 16:08h light: dark photoperiod). Germinated seed counts were conducted daily, and for this purpose, seeds with root lengths of at least 2 mm were considered germinated. Three germination parameters were determined using the following equations: Final Germination Percentage (FGP) (equation (S1)), Germination Rate (GR) (equation (S2)), and Mean Germination Time (MGT) (equation (S3)).

| $\boldsymbol{FGP (\%)=(}\frac{\boldsymbol{n}}{\boldsymbol{N}}\boldsymbol{) \times100}$ | (S1) |
| --- | --- |
| $\boldsymbol{GR (seed/day)=}\sum\boldsymbol{(}\frac{\boldsymbol{n}_{\boldsymbol{i}}}{\boldsymbol{t}_{\boldsymbol{i}}}\boldsymbol{)}$ | (S2) |
| $\boldsymbol{MGT (day)=}\frac{\sum\boldsymbol{(}\boldsymbol{n}_{\boldsymbol{i}}\boldsymbol{t}_{\boldsymbol{i}}\boldsymbol{)}}{\sum\boldsymbol{n}}$ | (S3) |

where:

*n =* The number of germinated seeds

*N =* The total number of seeds

$t_{i}$*=* The number of days from the start of the experiment to the end of the period

$n_{i}$*=* The number of germinated seeds on the i-th day

The analysis of variance and mean comparison (LSD) showed significant differences (p ≤ 0.001) in germination traits, including the Final Germination Percentage (FGP), Germination Rate (GR), and Mean Germination Time (MGT) (**Fig S1**). Thus, as shown in **Fig S1A**, 100% germination occurred in populations IR3329, IR5494, and IR2385. While population IR3776 had the lowest germination percentage at 30.66%. In terms of germination rate, populations IR3329, IR2385 and IR4457 displayed the highest rates at 20.88, 20.55, and 19.73 (seed/day), respectively, while population IR3776 had the lowest rate at 4.13 (seed/day) (**Fig S1B**). The quickest mean germination times were observed in populations IR3329, IR2385 and IR4457, with 1.34, 1.4 and 1.49 days, respectively, while the highest mean germination time for a duration of 2.6 days was observed in population IR2845 (**Fig S1C**).

| **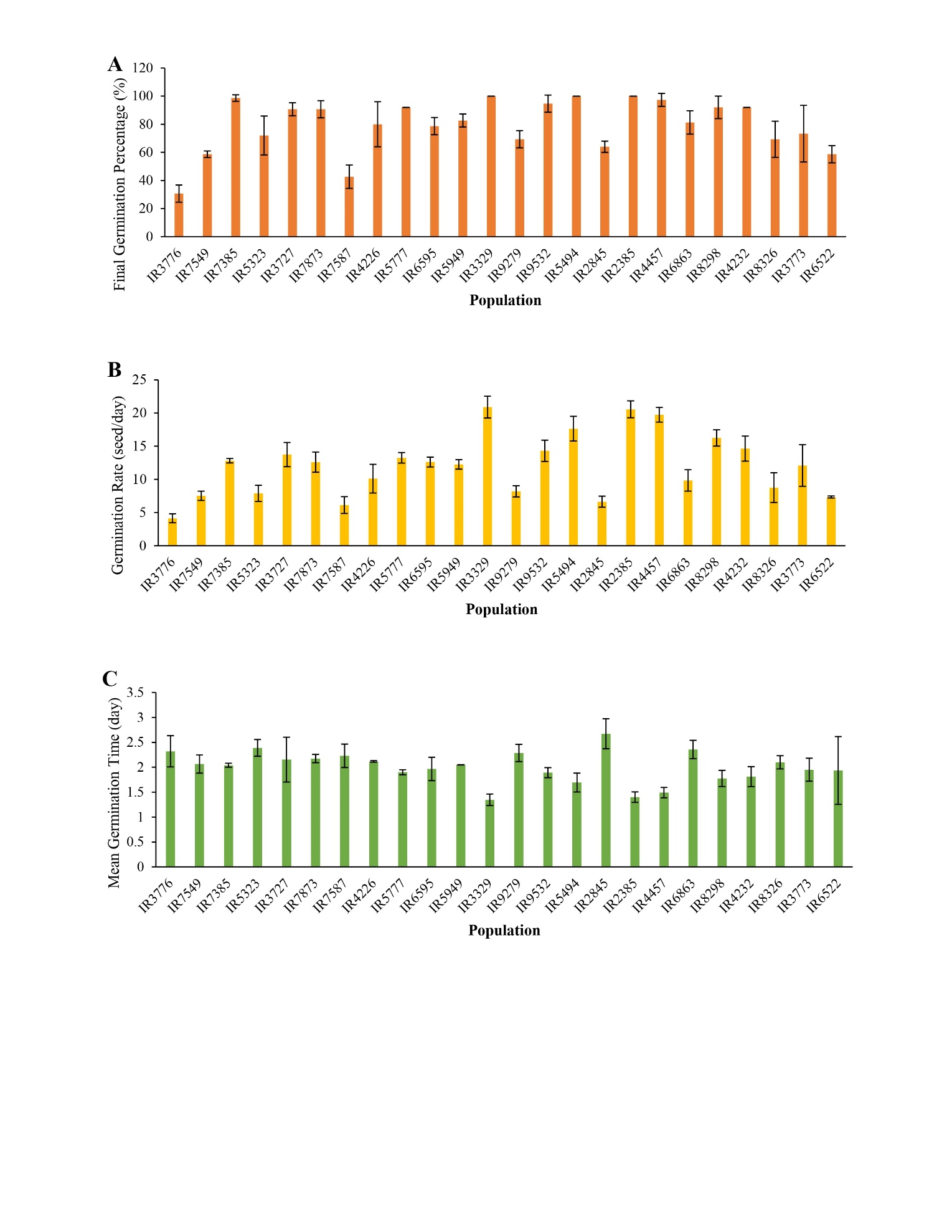**  **Fig S1.** Comparison of 25 native cannabis populations from Iran based on Final Germination Percentage (FGP) (A, LSD _0.01_= 17.5), Germination Rate (GR) (B, LSD _0.01_= 3.2) and Mean Germination Time (MGT) (C, LSD _0.01_= 0.5). |
| --- |
