# Supplementary material for: Characterization of indigenous populations of cannabis in Iran: a morphological and phenological study
Source: BMC Plant Biol. 2024 Feb 29;24:151. doi: 10.1186/s12870-024-04841-y (PMC10902964; doi:10.1186/s12870-024-04841-y)
Supplement: Supplementary file 2 — Supplementary Material 2 [file 12870_2024_4841_MOESM2_ESM.docx]

| LMI | FWF | DWF | TFW | TDW | HI | RGR | PT | SDH | HH | NNH | LIMTH | NLS | HGV | df | S.O.V |
| --- | --- | --- | --- | --- | --- | --- | --- | --- | --- | --- | --- | --- | --- | --- | --- |
| ^ns^ 184.24 | ^ns^ 2560 | ^ns^ 147 | ^ns^ 3440 | ^ns^ 1069.8 | ^ns^ 197.8 | ^ns^ 0.000042 | ^ns^ 1.03 | ^ns^ 3.25 | ^ns^ 452.3 | ^ns^ 36.71 | ^ns^ 10.72 | ^ns^ 36.81 | ^ns^ 34.99 | 2 | Block |
| ^***^106.1 | ^ns^ 1237 | ^ns^ 101.6 | ^***^ 7728 | ^**^ 1754.4 | ^**^ 441.1 | ^***^ 0.000258 | ^**^ 0.58 | ^***^ 16.97 | ^***^ 3107.1 | ^***^ 20.04 | ^***^ 27.04 | ^***^ 51.76 | ^***^ 42.665 | 24 | Population |
| 15.85 | 1386 | 131.2 | 2255 | 665.2 | 117.8 | 0.000011 | 0.26 | 3.19 | 387.8 | 3.33 | 4.829 | 7.47 | 225.21 | 48 | Error |
| 26.5 | 41.88 | 41.27 | 22.9 | 27.79 | 56.95 | 13.85 | 19.31 | 19.23 | 22.99 | 10.81 | 27.58 | 20.08 | 32.66 | - | CV (%) |

**Table S1** Analysis of variance (Mean Squares) for 14 morphological traits across 25 native cannabis populations in Iran, based on female plants.

ns, *, ** and *** indicate significant differences at not significant, P ≤ 0.05, 0.01 and 0.001, respectively. Abbreviations; DWF: Dry Weight of Flowers, FWF: Fresh Weight of Flowers, HGV: Height to GV Point, HH: Height in Harvest day, HI: Harvest Index, LIMTH: Length of Internode in the Middle Third of the main stem in Harvest day, LMI: Length of Main Inflorescence, NLS: Number of Lateral Shoot, NNH: Number of Nodes on the main stem in Harvest day, PT: Plant Type (1 to 4), RGR: Relative Growth Rate, SDH: Stem Diameter in Harvest day, TDW: Total Dry Weight, TFW: Total Fresh Weight.
