# Supplementary material for: Characterization of indigenous populations of cannabis in Iran: a morphological and phenological study
Source: BMC Plant Biol. 2024 Feb 29;24:151. doi: 10.1186/s12870-024-04841-y (PMC10902964; doi:10.1186/s12870-024-04841-y)
Supplement: Supplementary file 3 — Supplementary Material 3 [file 12870_2024_4841_MOESM3_ESM.docx]

| LMI | FWF | DWF | TFW | TDW | RGR | PT | SDH | HH | NNH | LIMTH | NLS | HGV | df | S.O.V |
| --- | --- | --- | --- | --- | --- | --- | --- | --- | --- | --- | --- | --- | --- | --- |
| ^ns^ 51.44 | ^ns^ 27.31 | ^ns^ 9.009 | ^ns^ 5894 | ^ns^ 929.3 | ^ns^ 0.000036 | ^ns^ 0.29 | ^ns^ 2.612 | ^ns^ 1.98 | ^ns^ 3.02 | ^ns^ 34.102 | ^ns^ 3.03 | ^ns^ 660.3 | 2 | Block |
| ^***^302.41 | ^***^ 292.78 | ^***^ 37.37 | ^***^ 13072 | ^***^ 2026.5 | ^***^ 0.000364 | ^***^ 1.108 | ^***^ 17.79 | ^***^ 3878.88 | ^***^ 44.85 | ^***^ 18.39 | ^***^ 79.15 | ^**^ 549.7 | 24 | Population |
| 24.35 | 123.25 | 13.25 | 1795 | 255.3 | 0.000016 | 0.27 | 1.67 | 312.05 | 2.66 | 5.85 | 9.82 | 291.1 | 48 | Error |
| 32.51 | 45.71 | 44.32 | 49.28 | 46.8 | 15.28 | 25.16 | 22.5 | 24.89 | 14.55 | 25.06 | 25.46 | 36.84 | - | CV (%) |

**Table S2** Analysis of variance (Mean Squares) for 13 morphological traits across 25 native cannabis populations in Iran, based on male plants.

ns, *, ** and *** indicate significant differences at not significant, P ≤ 0.05, 0.01 and 0.001, respectively. Abbreviations; DWF: Dry Weight of Flowers, FWF: Fresh Weight of Flowers, HGV: Height to GV Point, HH: Height in Harvest day, HI: Harvest Index, LIMTH: Length of Internode in the Middle Third of the main stem in Harvest day, LMI: Length of Main Inflorescence, NLS: Number of Lateral Shoot, NNH: Number of Nodes on the main stem in Harvest day, PT: Plant Type (1 to 4), RGR: Relative Growth Rate, SDH: Stem Diameter in Harvest day, TDW: Total Dry Weight, TFW: Total Fresh Weight.
