# Supplementary material for: Characterization of indigenous populations of cannabis in Iran: a morphological and phenological study
Source: BMC Plant Biol. 2024 Feb 29;24:151. doi: 10.1186/s12870-024-04841-y (PMC10902964; doi:10.1186/s12870-024-04841-y)
Supplement: Supplementary file 4 — Supplementary Material 4 [file 12870_2024_4841_MOESM4_ESM.docx]

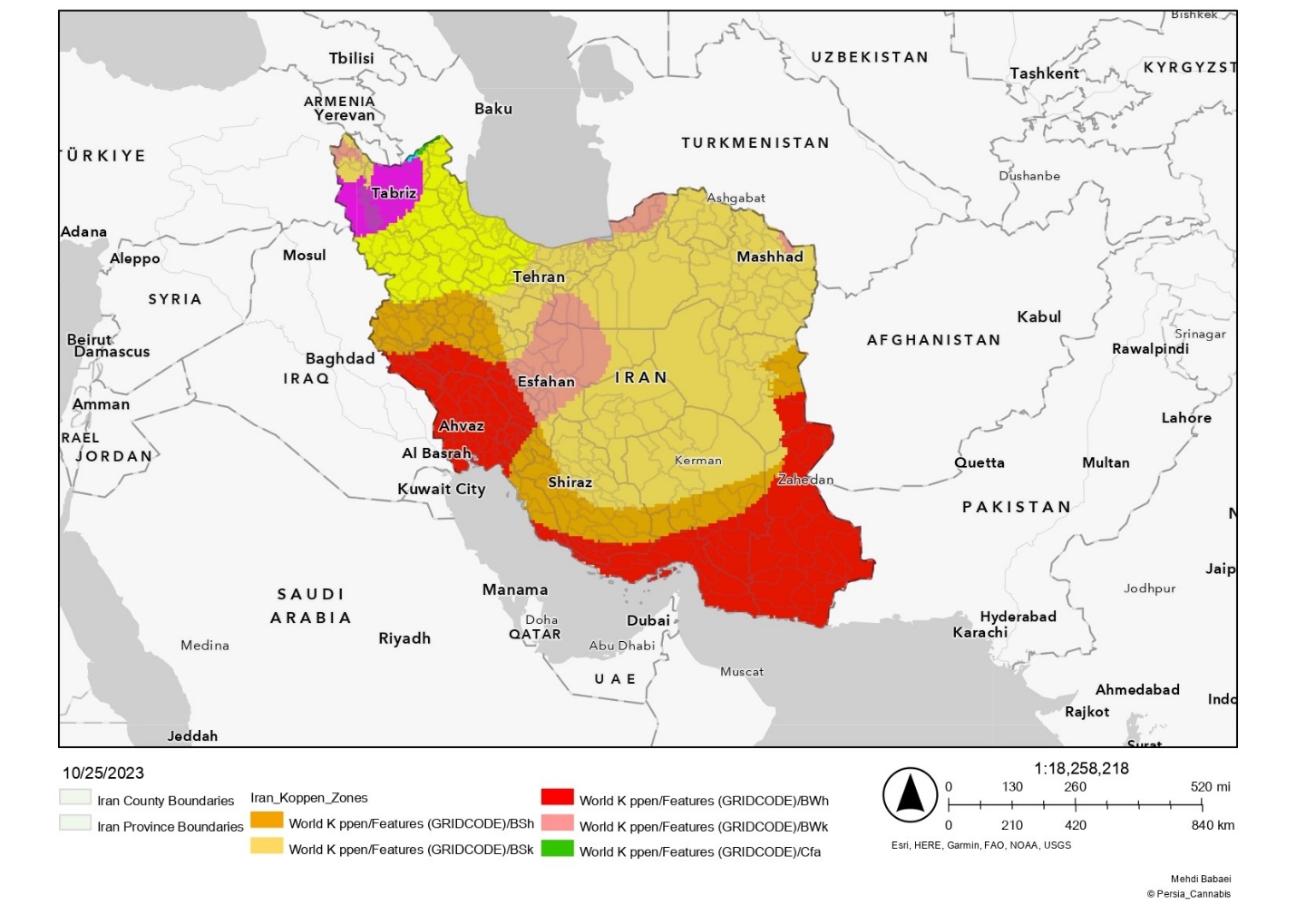


**Fig S2.** Map of exploratory sites for indigenous populations of cannabis in Iran based on five climatic zones (BSh: arid, steppe, hot; BSk: arid, steppe, cold; BWh: arid, desert, hot; BWk: arid, desert, cold; Cfa: temperate, without dry season, hot summer). This map is licensed under the Creative Commons Attribution-ShareAlike 3.0 Unported License (CC BY-SA 3.0). Changes to the original material have been made using ArcGIS Online (Developed by Esri) to update geographical features and the addition of provincial and national borders to enhance geographical detail [1, 2].

**References**

1. Kottek M, Grieser J, Beck C, Rudolf B, Rubel F. World map of the Köppen-Geiger climate classification updated. 2006.

2. Köppen W. Versuch einer Klassifikation der Klimate, vorzugsweise nach ihren Beziehungen zur Pflanzenwelt. Geogr Z. 1900;6 11. H:593–611.
