# Supplementary material for: Characterization of indigenous populations of cannabis in Iran: a morphological and phenological study
Source: BMC Plant Biol. 2024 Feb 29;24:151. doi: 10.1186/s12870-024-04841-y (PMC10902964; doi:10.1186/s12870-024-04841-y)
Supplement: Supplementary file 5 — Supplementary Material 5 [file 12870_2024_4841_MOESM5_ESM.docx]

**Soil sample preparation and laboratory analysis procedure**

Ten different soil mixtures were selected, blended, and placed in an open space for 72 hours to dry. Subsequently, particles were separated using 2-millimeter sieves, and the samples were submitted to the Soil Science Laboratory at Ferdowsi University of Mashhad, Iran, for soil analysis using standard laboratory methods.

**Table S3** Physical and chemical features of the soil.

| Texture | pH | EC  (dsm^-1^) | OC (%) | N  (ppm) | P  (mg kg^-1^) | K  (mg kg^-1^) | Fe  (ppm) | Ca  (mEq/lit) | Mg  (mEq/lit) |
| --- | --- | --- | --- | --- | --- | --- | --- | --- | --- |
| Sandy loam | 8.04 | 1.56 | 2.59 | 1850 | 18.9 | 400 | 4.21 | 50 | 37.5 |
