# Supplementary material for: Characterization of indigenous populations of cannabis in Iran: a morphological and phenological study
Source: BMC Plant Biol. 2024 Feb 29;24:151. doi: 10.1186/s12870-024-04841-y (PMC10902964; doi:10.1186/s12870-024-04841-y)
Supplement: Supplementary file 6 — Supplementary Material 6 [file 12870_2024_4841_MOESM6_ESM.docx]

| Nutrient | Vegetative stage | | | | Reproductive stage | | | |
| --- | --- | --- | --- | --- | --- | --- | --- | --- |
|  | Final nutrient concentration delivered (ppm) ^a^ | Fertilizer sources ^b^  (ppm nutrient provided) | | | Final nutrient concentration delivered (ppm) ^a^ | Fertilizer sources ^b^  (ppm nutrient provided) | | |
|  |  | Ammonium Phosphate ((NH_4_)_3_PO_4_) ^c^ | Calcium Nitrate  (Ca (NO_3_)_2_) ^d^ | Potassium Nitrate  (KNO_3_) ^e^ |  | Ammonium Phosphate ((NH_4_)_3_PO_4_) ^c^ | Calcium Nitrate  (Ca (NO_3_)_2_) ^d^ | Potassium Nitrate  (KNO_3_) ^e^ |
| N | 200 | 24 | 120 | 56 | 200 | 49 | 81 | 70 |
| P | 120 | 120 | - | - | 250 | 250 | - | - |
| K | 200 | - | - | 200 | 250 | - | - | 250 |
| Ca | 233 | - | 233 | - | 158 | - | 158 | - |
| Total | 753 | 144 (197) | 353 (889) | 256 (435) | 858 | 299 (409) | 239 (600) | 320 (543) |

**Table S4** Final nutrient concentrations and sources in different growth stages.

^a^ 1 ppm = 1 mg/liter.

^b^ Nutrient composition varies by source, affecting the amount needed for target concentrations.

^c^ 12% Nitrogen (N) and 61% Phosphorus (P).

^d^ 26.3% Calcium (Ca) and 13.5% N.

^e^ 46% Potassium (K) and 13% N.

Note: Values in parentheses represent the actual amounts used, adjusted for purity.
