# Supplementary material for: Characterization of indigenous populations of cannabis in Iran: a morphological and phenological study
Source: BMC Plant Biol. 2024 Feb 29;24:151. doi: 10.1186/s12870-024-04841-y (PMC10902964; doi:10.1186/s12870-024-04841-y)
Supplement: Supplementary file 7 — Supplementary Material 7 [file 12870_2024_4841_MOESM7_ESM.docx]

**Table S5** Evaluation metrics for spatial analysis model adjusting variations among plants in two-dimensional space across columns and rows.

|  | **Female** | | | | **Male** | | | |
| --- | --- | --- | --- | --- | --- | --- | --- | --- |
| **Trait** | **MSE** | **RMSE** | **NRMSE** | **CV%** | **MSE** | **RMSE** | **NRMSE** | **CV%** |
| LMI (cm) | 4.8 | 2.2 | 0.1 | 8.2 | 13.8 | 3.7 | 0.11 | 11.2 |
| PT (1 to 4) | 0.1 | 0.3 | 0.1 | 12 | 0.16 | 0.4 | 0.13 | 13.7 |
| SDH (mm) | 1 | 1 | 0.07 | 7.1 | 0.77 | 0.8 | 0.07 | 7.5 |
| HH (cm) | 154 | 12.4 | 0.08 | 8 | 127.9 | 11.3 | 0.07 | 7.3 |
| NNH (n) | 1.9 | 1.3 | 0.04 | 4.8 | 0.9 | 0.9 | 0.03 | 3.5 |
| LIMTH (cm) | 1.9 | 1.4 | 0.11 | 11.1 | 0.8 | 0.9 | 0.07 | 7 |
| NLS (n) | 3.3 | 1.8 | 0.07 | 7.6 | 5.3 | 2.3 | 0.1 | 10.3 |
| HGV (cm) | 117 | 10.8 | 0.18 | 18.6 | 116.7 | 10.8 | 0.2 | 20.2 |
| FWF (gr) | 418.3 | 20.4 | 0.23 | 23.1 | 70.7 | 8.4 | 0.3 | 29 |
| DWF (gr) | 57.7 | 7.5 | 0.28 | 28.3 | 6.9 | 2.6 | 0.25 | 25.5 |
| TFW (gr) | 1229.8 | 35 | 0.12 | 12.5 | 1148.6 | 33.8 | 0.22 | 22.3 |
| TDW (gr) | 200.3 | 14.1 | 0.12 | 12.2 | 153.5 | 12.3 | 0.2 | 19.9 |
| HI (%) | 24.5 | 4.9 | 0.18 | 18.8 | - | - | - | - |
| RGR (g.g^-1^.day^-1^) | 0 | 0.001 | 0.02 | 2.7 | 0 | 0.003 | 0.04 | 4 |
| GVP (day) | 35.3 | 5.9 | 0.08 | 8.2 | 14.6 | 3.8 | 0.05 | 5.6 |
| SFFI (day) | 9.9 | 3.1 | 0.04 | 4.4 | 15.9 | 3.9 | 0.05 | 5.5 |
| SFFP (day) | 17 | 4.1 | 0.06 | 5.9 | 21.1 | 4.5 | 0.06 | 6.5 |
| SF10I (day) | 84.6 | 9.2 | 0.08 | 8.2 | 42.2 | 6.5 | 0.07 | 6.8 |
| SF10P (day) | 84 | 9.1 | 0.08 | 8.3 | 46.6 | 6.8 | 0.07 | 7.3 |
| FT50I (day) | 36.5 | 6 | 0.04 | 4.5 | 44.3 | 6.6 | 0.06 | 6.4 |
| OFS (day) | - | - | - | - | 66.3 | 8.1 | 0.08 | 8.1 |

Abbreviations; LMI: Length of Main Inflorescence, PT: Plant Type (1 to 4). SDH: Stem Diameter in Harvest day, HH: Height in Harvest day, NNH: Number of Nodes on the main stem in Harvest day, LIMTH: Length of Internode in the Middle Third of the main stem in Harvest day, NLS: Number of Lateral Shoot, HGV: Height to GV Point, FWF: Fresh Weight of Flowers, DWF: Dry Weight of Flowers, TFW: Total Fresh Weight, TDW: Total Dry Weight, HI: Harvest Index, RGR: Relative Growth Rate, GVP: GV Point, SFFI: Start Flower Formation Time in Individuals, SFFP: Start Flower Formation Time in 50% Population, SF10I: Start 10% Flowering Time in Individuals (10 % of bracts formed), SF10P: Start 10% Flowering Time in 50% Population (10 % of bracts formed), FT50I: Flowering Time 50% in Individuals (50 % of bracts formed), OFS: First Opened Staminate Flowers.
